# A spore quality–quantity tradeoff favors diverse sporulation strategies in *Bacillus subtilis*

**DOI:** 10.1038/s41396-020-0721-4

**Published:** 2020-07-28

**Authors:** Alper Mutlu, Charlotte Kaspar, Nils Becker, Ilka B. Bischofs

**Affiliations:** 1 Max-Planck-Institute for Terrestrial Microbiology, D-35043 Marburg, Germany; 2 BioQuant Center of Heidelberg University, D-69120 Heidelberg, Germany; 3 Center for Molecular Biology (ZMBH), Heidelberg University, D-69120 Heidelberg, Germany; 4 Division of Theoretical Systems Biology, German Cancer Research Center (DKFZ), D-69120 Heidelberg, Germany

**Keywords:** Bacterial genetics, Biological sciences, Bacterial evolution

## Abstract

Quality–quantity tradeoffs govern the production of propagules across taxa and can explain variability in life-history traits in higher organisms. A quality–quantity tradeoff was recently discovered in spore forming bacteria, but whether it impacts fitness is unclear. Here we show both theoretically and experimentally that the nutrient supply during spore revival determines the fitness advantage associated with different sporulation behaviors in *Bacillus subtilis*. By tuning sporulation rates we generate spore-yield and spore-quality strategists that compete with each other in a microscopic life-cycle assay. The quality (yield) strategist is favored when spore revival is triggered by poor (rich) nutrients. We also show that natural isolates from the gut and soil employ different life-cycle strategies that result from genomic variations in the number of *rap-phr* signaling systems. Taken together, our results suggest that a spore quality–quantity tradeoff contributes to the evolutionary adaptation of sporulating bacteria.

## Introduction

Endospores of the model organism *Bacillus subtilis* have been isolated from almost every niche on Earth, but primarily from the soil and from the gut flora of organisms that (partially) feed directly from the ground, including chickens, pigs, mice, and also humans [1]. *B. subtilis* sporulates both in the rhizosphere environment of soil [2] and in the gut [3–5], and spores also revive there [2, 5, 6]. Diverse natural isolates have been characterized, and it was noted repeatedly that their sporulation kinetics is strikingly variable [4, 7, 8]. This suggests that sporulation timing is an evolvable and highly variable life-cycle trait.

By making spores, *B. subtilis* adapts to fluctuations in nutrient availability [9]; in addition, the spore protects the genome from stress [10], which could be advantageous when transitioning into harsh environments [11]. Surprisingly, diverse feed-and-starvation cycles applied to mutagenized *B. subtilis* laboratory populations have failed to enrich for mutants with altered sporulation behavior [12]. Moreover, laboratory evolution experiments involving exposure to heat and intended to select for higher sporulation efficiency have been unsuccessful [13], although sporulation is readily lost when not selected for [14]. The resilience of *B. subtilis* in the face of adjustments of its sporulation kinetics in the laboratory is in sharp contrast to the variability seen in nature. How diverse sporulation strategies arise thus remains an open question, which is both of fundamental significance and of potential interest for commercial spore production of this beneficial bacterium [15, 16].

Laboratory evolution [13] and selection experiments [12] have generally considered sporulation as an isolated trait, as sporulation in *B. subtilis* has conventionally been studied separately from spore revival. The initiation of each process is controlled by a distinct molecular network [17, 18]. Sporulation timing is regulated by the activation of the master regulator Spo0A via the sporulation phosphorelay [19, 20]. Under starvation conditions, sporulation kinases induce a flux of phosphoryl groups toward Spo0A, which is counteracted by sporulation inhibitors, among them a subset of *rap*-family phosphatases [21–23]. Spore revival is triggered when new nutrients become available that trigger germination (in which the spore rehydrates and dismantles its protective structure) and support spore outgrowth (in which the spore reactivates macromolecular synthesis and eventually escapes from the spore coat upon resuming vegetative growth) [24–26].

The paradigm that sporulation and spore revival act as independent life-cycle traits has been challenged in recent years, as sporulation conditions, spore age and variation in sporulation timing can affect spore revival [27–30]. We recently found that sporulation timing controls the spore’s revival capacity independently of spore age. A delay in sporulation timing negatively affects sporulation traits that are relevant for successful spore revival and thus causes a decrease in spore quality. At the same time a delay in sporulation timing allows for additional cell divisions to take place and thus it increases the number of produced spores. This results in a tradeoff between spore quality and spore quantity [30]. Although little is yet known about the underlying spore-quality determinants, spore quality is in part mediated by an enzymatic marker, alanine dehydrogenase (Ald). Ald was found to be a limiting resource during starvation, which is allocated to spores and controls spore revival by the nutrient–germinant l-alanine [30].

Quality–quantity tradeoffs [31] are well known to govern the production of propagules across taxa, including plants [32], arthropods [33], fish [34], birds [35], and mammals [36, 37], although the underlying molecular mechanisms are mostly unknown. Such tradeoffs are an integral part of life-history theory in higher organisms [38, 39] and have been proposed to drive adaptations to different ecological niches [33, 40, 41]. However, this kind of life-history tradeoff need not necessarily have an impact on overall fitness. In birds, fitness is barely affected, if at all [35], and whether the tradeoff is relevant to fitness in humans is disputed [37]. By analogy, for sporulating *B. subtilis* bacteria, a change of life-cycle strategy might not affect fitness since gains in spore quantity come at the cost of reductions in spore quality (and vice versa) [30].

Here we ask whether a spore quality–quantity tradeoff affects the fitness of *B. subtilis*. Guided by a theoretical model of the spore quality–quantity tradeoff in *B. subtilis*, we show experimentally that the nutrient supply during spore revival determines the fitness advantage associated with different sporulation behaviors, in both a synthetic model with a tunable life-cycle strategy and in natural isolates from the soil and the chicken gut, respectively. Our data support the idea that a spore quantity–quality tradeoff is relevant for adaptation to different ecological niches, and suggest that spore-revival conditions may contribute to the evolution of sporulation traits.

## Materials and methods

### Strains and plasmids

*B. subtilis* strains were derived from 1A700 (W168), BSP1, and 3A36 (PS216). *E. coli* DH5α was used for cloning. All strains, plasmids and oligonucleotides used in this study are listed in Tables S1, S2, and S3, respectively. Construction of strains and plasmids for Supplementary Data are described in the Supplementary Material.

#### Plasmid construction

pDR111-*mCherry* (EIB424): the *mCherry* coding region was amplified from the genomic DNA of *B. subtilis* BIB182 (Michael Elowitz, CalTech) using the primers MA24 (SalI) and MA25 (SphI), and inserted into the multiple cloning site of the pDR111 vector (D. Rudner, Harvard University, Boston) by restriction-enzyme ligation cloning. The plasmid was sequenced with the primers SONSEQ18 and SONSEQ19.

#### Strain construction

Strains were constructed by transforming *B. subtilis* with the indicated ectopic integration vectors listed in Table S2 using standard protocols [9]. All fluorescent reporters are present in single copy in the chromosome and are expressed from an ectopic locus (e.g. *amyE*). Corrcect locus integration and the absence of single cross-over events were verified by PCR using appropriate primers listed in Table S3.

### Media and solutions

Strains were grown under aeration (unless otherwise noted) in LB medium (Lennox version [42]), in casein hydrolysate (CH) [9], sporulation medium (SM [9]), or on agarose pads supplemented with modified SM (SM with a reduced level of glutamate (10%)) or SM* (SM with a reduced level of glutamate (10%) and 1 mM l-alanine) [30] at 37 °C, unless otherwise noted. The appropriate antibiotics and amino acids were added as required [chloramphenicol (5 µg ml^−1^), spectinomycin (100 µg ml^−1^), neomycin (1 µg ml^−1^), erythromycin (1 µg ml^−1^) and kanamycin (10 µg ml^−1^)]. For cultivation of *B. subtilis* B168, l-tryptophan was added at 20 µg ml^−1^ and 22 µg ml^−1^ to CH and SM* media, respectively. Nutrient solutions for spore-revival experiments were performed with l-alanine (100 mM), LB and AGFK (19.8 mM l-asparagine, 33.6 mM d-glucose, 33.6 mM d-fructose and 60 mM KCl), unless otherwise noted. Inducer solutions for gene expression contained 1 mM isopropyl-β-D-thiogalactopyranoside (IPTG).

### Life-cycle assay

The life-cycle assay was used to induce sporulation and spore revival as previously described [30], with the modifications described below. In brief, cells were grown overnight at 37 °C in liquid CH media with appropriate selection. Next, cells were re-inoculated into fresh CH medium (without selection) and grown to OD_600nm_ = 0.8–1 and then resuspended in SM* media to a standardized OD_600nm_ = 0.1. Competing strains were mixed in a 1:1 ratio, and 4 µl of the cell suspension were used to inoculate a defined 1.5% agarose pad (9 mm diameter, 1 mm height) containing SM* media mixed with IPTG, if required. The pads were then stamped into a 24-well glass-bottomed SensoPlate (Greiner Bio-One, Germany). This inoculation procedure confines cells to a defined imaging plane and provides a suitable area cell density for analysis. For experiments involving co-cultures of natural isolates (and their derivatives) we empirically found that we could reduce their tendency to form multi-layered colonies by increasing the overall cell numbers on the pad. This was accomplished by distributing cells on opposite gel surfaces: the side facing the glass bottom was inoculated as before to maintain the optimal cell density per unit area for analysis. An additional 2 µl of the cell suspension aliquots was placed on the opposite side, i.e., the pad surface that faces the air after stamping pads into the well. Pads were imaged in a microscope-associated environmental control chamber held at 36.5 °C. Positions were manually selected to track the development of cells/spores from both strains in the same field of view. The starvation response was monitored by time-lapse microscopy for 4 days until sporulation was complete. To induce spore revival 10 µl of the indicated nutrient solution was added to the back side of the pad.

### Time-lapse microscopy

Imaging was performed on an automated DeltaVision Elite Imaging System (Applied Precision, Issaquah, WA, USA) with Resolve3D SoftWorx-Acquire Version 6.1.1 Release 5, as described before [30]. In brief, cells and spores were imaged with a ×40/NA = 0.95 air objective and a 1.6-fold auxiliary magnification lens. Fluorescent reporters were excited using the following settings and filter sets (excitation, emission): CFP: 0.25 s with 100% excitation (438 nm/24, 470 nm/24), mCherry: 0.1 s with 100% excitation (575 nm/25, 632 nm/60) and a CFP/YFP/mCh dichroics (reflection bands: 400–45, 496–52, 558–594 nm; transmission bands: 463–487, 537–550, 602–648 nm). The UV filter was engaged to minimize phototoxicity. Images were recorded every 20 min with a PCO Edge sCMOS camera using 1024 × 1024 pixels.

### Data analysis

Time-lapse movies were analyzed with the help of the Fiji software [43]. Movies for which both the starvation and the revival response of at least one colony from each strain were completely captured in the field of view were analyzed quantitatively.

#### Spore yield

To determine the spore yield, we counted the number of spores *N*_*s*_ in spore microcolonies after 4 days of starvation. At this stage, sporulation was complete in most microcolonies and few, if any, vegetative cells were still present. We accounted for differences in spore yield resulting from variations in initial population size. Since rod-shaped cells grow in length, the initial population size was approximated by the cumulative length *L*_*c*_ of all cells that seed a spore microcolony. The spore yield per “standard” cell is *Y* = *βN*_*s*_ with the biomass normalization factor $\beta = \frac{{L_1}}{{L_c}}$. Here, *L*_1_ is the typical length of a single cell as determined by averaging the cell length of 40 single cells in the first frame of the sporulation movie.

#### Spore-revival fitness

To quantify the revival fitness *f* = ln(*S*) we counted the number of reviving spores *N*_r_ in spore microcolonies as a measure of the revival success per standard cell *S* = *βN*_r_, where *β* is the biomass normalization factor. A spore was scored as revived, when a visible crack appeared in the germinated spore, signaling the imminent emergence of a cell. We counted all events of outgrowing spores within a period of either 12 or 2 h post stimulation with poor and rich nutrients, respectively. At this time, the proliferating cells (resulting from revived spores) typically overgrow the spore colony.

#### Statistics

For each experiment we analyzed at least *N* ≥ 8 microcolonies (movies) from *n*_r_ ≥ 2 technical replicates. A two-tailed Mann–Whitney *U* test to determine the statistical significance of the differences in the observed distributions.

## Results

### Different life-cycle strategies are favored in the spore quality–quantity tradeoff model

To explore how the processes of sporulation and revival may interact to modulate overall fitness, we first considered a theoretical model of sporulation and revival. The model is informed by recent findings on a spore quality–quantity tradeoff in *a B. subtilis* laboratory strain [30], and was extended to incorporate plausible assumptions for the mechanism of revival based on additional experimental data that investigate the role of Ald in the metabolism of the germinated spore (Fig. S1, see Supplementary text for details). We focus on environmental conditions with long starvation periods, in which case spores are essential for survival, as cells that do not differentiate into dormant spores will eventually die (Fig. S2a). When new nutrients become available, spores revive and the outgrowing spores establish a new population of growing and dividing cells. To quantify the contribution of one full cycle of sporulation and revival to reproductive fitness, we define revival success *S* = *N*_r_/*N*_v_ as the ratio of the number of reviving spores *N*_r_ to the number of vegetative cells *N*_v_ that entered starvation, and revival fitness *f* = ln(*S*), which serves as a measure of the relative increase (or decrease) in the population size per starvation-revival cycle. The revival fitness is neutral (*f* = 0) if, during starvation, each cell differentiates into a single spore that revives successfully upon receiving new nutrients. If some spores fail to revive, the fitness becomes negative (*f* < 0). Conversely, a bacterium that is able to delay sporulation, continuing to replicate to finally generate more than one spore, can compensate for spore-revival failures, resulting in neutral or positive revival fitness (*f* > 0).

In order to increase the revival fitness *f* bacteria could either increase spore yield *Y* or enhance spore quality *q* to boost the chances of successful revival. The nature of a quality–quantity tradeoff implies that quality and yield are related to each other, i.e.:

1 $$Y = \int_{0}^{\infty}\! \sigma \left(q\right)dq,$$

here, *σ*(*q*) is the number density of spores of quality *q* produced by a single cell, as determined by the sporulation strategy given the constraints imposed by the quality–quantity tradeoff. Generic models of quality–quantity tradeoffs in higher organisms assume that the resources available for investment in the generation of offspring are inherently limited. This constrains options for the life-history strategy to either devoting resources to making few offspring of high quality or many of low quality [31]. By analogy, a fixed resource (e.g., amount of a specific protein such as Ald) present in a starving bacterium will be shared equally among the progeny upon cell division, which will progressively diminish the available resources for enhancing spore quality. The enzyme Ald is a limited resource, and thus enzyme levels in spores decay exponentially with sporulation timing (Fig. S1a, see Supplementary text for details). We therefore assume that spore quality *q* decreases exponentially with each cell division that preceded sporulation (Fig. S2a). Thus, a low sporulation rate produces many spores of low quality but with a high overall yield; we call such a strategy a “yield strategy.” Conversely, a high sporulation rate will increase the number of high-quality spores but with lower overall yield; we refer to this a “quality strategy” (Fig. S2b).

Whether or not changes in the sporulation strategy are adaptive and thus provide a fitness advantage depends on environmental conditions during spore revival. Upon provision of nutrients, spores revive with some probability *P*_*m*_(*q*), which depends on both the spore quality *q* and the revival conditions, summarized by *m*. The resulting overall revival fitness is then:

2 $$f = {\mathrm{ln}} \int_{0}^{\infty} \sigma \left( q \right)P_{\mathrm{m}}\left( q \right)dq.$$

The physiology of spore revival suggests that *P*_*m*_ depends on an adequate level of metabolism in the germinated spore: in “poor” media, Ald-dependent metabolism of the nutrient—and germinant—l-alanine is required to power spore outgrowth, while in “rich” media other substrates generate an additional contribution [30, 44] (see Supplementary text and Fig. S1d, e), which we assume to be independent of the quality *q* (Fig. S3). Thus in rich media, all spores revive, regardless of their quality and thus a yield strategy is the better option. In contrast, in poor media fitness is optimized by a quality strategy. Notably for intermediate media, fitness is largely unaffected by changes in sporulation rate (Fig. S4). In sum, this analysis suggests that the nutrient supply during revival—specifically rich and poor media, respectively—could select for different sporulation strategies.

### The nutrient supply during revival discriminates between different sporulation strategies

To experimentally determine the revival fitness of sporulating *B. subtilis* bacteria, we utilized a microscopic life-cycle assay [30]. After growth in liquid CH medium, bacteria are subjected to a starvation period of about 4 days by placing individual cells on a hydrogel pad containing SM*, where they first grow into microcolonies and then sporulate (or die). Upon provision of nutrients to the pad, spores revive and the outgrowing cells grow and proliferate (until nutrients are depleted again). The addition of the nutrient–germinant l-alanine should stringently select for outgrowth of high-quality spores in the given environmental context [30, 44], while LB should be less selective since it provides a rich mixture of nutrients [42] to the spore. For simplicity we denote these unequal revival settings as nutrient-poor and nutrient-rich, respectively.

Thus, the revival fitness *f* can be determined from the revival success *S* by counting the number of outgrowing spores generated by a single “standard” cell in one starvation-revival cycle. Since the population size that seeds a spore microcolony affects the numbers of spores generated, we accounted for effects of initial biomass variation when determining the values of *Y* and *S*, respectively (see “Materials and methods” for details). One standard cell of the *B. subtilis* 168 laboratory strain (BIB1094) produced on average $\bar Y = 78$ spores, albeit with substantial variation from colony to colony (Fig. S5a). When revival occurred in a nutrient-rich environment, many spores revived successfully, while only a few high-quality spores grew in a nutrient-poor environment (Fig. S5b).

**Fig. 1:**
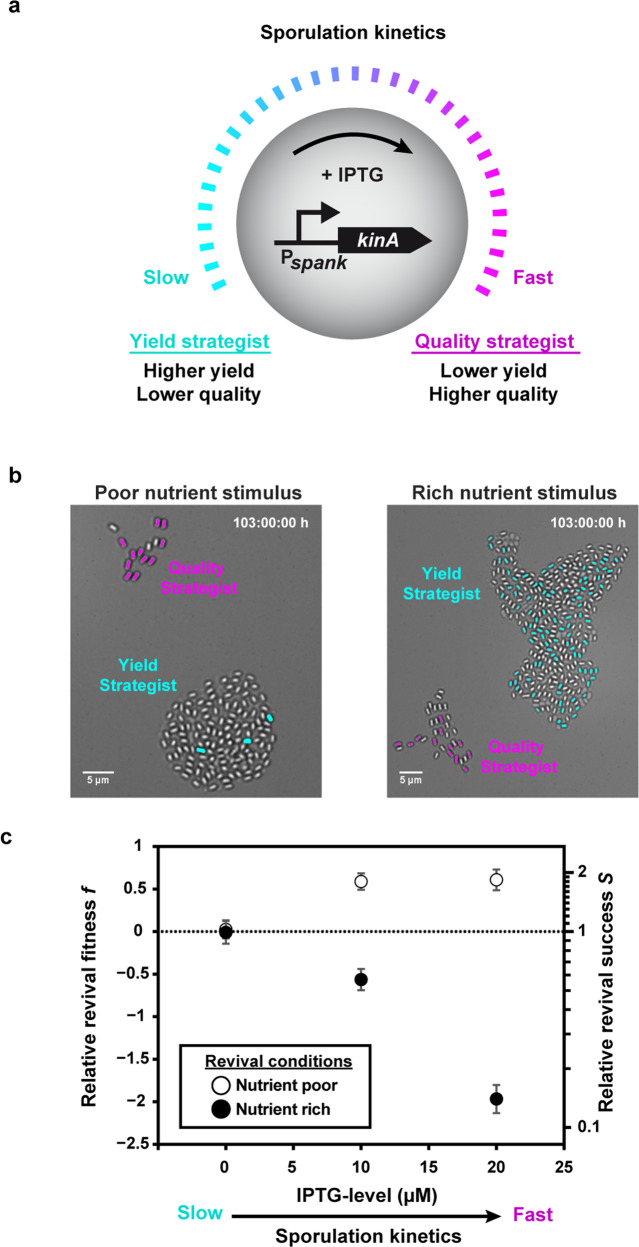
Fitness of a synthetic quality strategist vs. yield strategists measured under different revival conditions. **a** Schematics showing the synthetic model system that was used to alter the life-cycle strategy of a *Bacillus subtilis* laboratory strain B168 in response to nutrient fluctuations. **b** A synthetic quality strategist (KinA ↑, magenta) competed against the yield strategist (wild type, cyan) under fluctuating nutrient conditions in a microscopic life-cycle assay in the presence of 20 µM IPTG. The wild-type cells carry an IPTG-inducible fluorescent reporter to distinguish them from the synthetic strain. The micrographs show brightfield images of the resulting spore colonies for each strain. The reviving spores are false colored in magenta (KinA ↑, BIB1332) and cyan (wild type, BIB1092). Left: supply of “poor” medium (100 mM l-alanine). Right: supply of rich medium (LB). See Movies S1 (10.5446/42828) and S2 (10.5446/42829) for full sporulation and revival dynamics. See “Materials and methods” for details. **c** Relative revival success *S* = *S*_KinA_/*S*_wt_ in response to the different nutrient stimuli as a function of IPTG levels. Empty and full circles: 100 mM l-ala and LB nutrient stimulation, respectively. *N* ≥ 9 colonies per condition from *n* ≥ 2 technical replicates.

According to our model, different revival conditions should favor different life-cycle strategies that invest in either spore quantity or spore quality. To experimentally generate alternative life-cycle strategies, we used a synthetic model system derived from the B168 laboratory strain. Overexpression of the sporulation kinase KinA from an IPTG-inducible promoter increases the sporulation probability and accelerates the kinetics of sporulation [45]. The sporulation rate directly controls the number of spores that are produced in response to a nutrient downshift: an accelerated sporulation rate decreases spore yield. Accelerated sporulation also shifts the quality distribution as approximated by the levels of the spore-quality marker Ald in the resulting spores (Fig. S1b, c), which enhances their capacity to grow out in response to l-alanine in a nutrient-poor environment [30]. To summarize, increasing IPTG levels in a KinA-inducible synthetic strain causes the spore yield to decrease, while the capacity for spore revival increases—and a “yield strategist” is thus transformed into a “quality strategist” (Fig. 1a).

To assess how a change in the life-cycle strategy affects fitness under rich vs. poor revival conditions, the synthetic strain was competed with a fluorescently labeled wild-type strain in the presence of different levels of IPTG. The wild-type strain exhibits the spore yield and spore-revival fitness of the uninduced synthetic strain, and thus serves as a reference for the “yield strategy” in these experiments (Fig. S5). With IPTG induction the synthetic strain sporulates faster and its revival frequency in response to l-alanine increases; hence, it represents the quality strategist. At maximal induction the spore yields of the quality strategist and the yield strategist were strikingly different (Fig. S6). Upon provision of LB, many spores produced by either strain revived successfully (Fig. 1b, right). As a result, the revival fitness was mainly determined by the spore yield. Thus, in a nutrient-rich environment the yield strategist was fitter than the quality strategist (even though LB did not elicit the full potential of yield-driven fitness since germination was incomplete, as judged by the number of spores that lost their refractivity, see Movie S1, 10.5446/42828). In contrast, almost all spores germinated in the presence of the nutrient–germinant l-alanine, but far fewer spores succeeded in outgrowth as spore quality became limiting (Movie S2, 10.5446/42829). Thus, under nutrient-poor conditions, the quality strategist had a fitness advantage over the yield strategist (Fig. 1b, left). We quantified the relative revival success *S* = *S*_KinA_/*S*_wt_ and the relative revival fitness *f* = *f*_KinA_/*f*_wt_ as a function of IPTG levels for nutrient-rich and -poor conditions (Fig. 1c). Under nutrient-poor conditions that select for high-quality spores, the relative revival success increased up to twofold, resulting in a moderate fitness advantage for the quality strategist (Fig. 1c, empty circles). In the presence of rich nutrients, relative revival success decreased by up to tenfold when sporulation was accelerated, resulting in a strong fitness advantage for the yield strategist (Fig. 1c, filled circles).

As an additional control, we developed a co-culture sporulation assay in a shaken liquid culture by mixing fluorescently labeled wt cells and the IPTG-inducible KinA strain at a 1:1 ratio (Supplementary methods, Fig. S7a). As expected, accelerated sporulation induced by IPTG results in a lower frequency of KinA spores in the overall spore population (Fig. S7b) and concomitantly increased their capacity for l-alanine-dependent spore outgrowth (Fig. S7c). This overall gain in spore quality is reflected in a shift in the distribution of the Ald spore quality marker to higher levels (Fig. S1c). We next monitored the formation of microcolony-forming units (mCFUs) from the spore mixture by time-lapse microscopy (Movies S3, 10.5446/46818 and S4, 10.5446/46819). Although spores of the synthetic KinA strain were in the minority, they accounted for the majority of mCFUs in a nutrient-poor environment. In contrast, in a very rich nutrient environment (LB plus a mixture of the germinant l-alanine and the germinant mixture AGFK to boost germination [24, 25] and thereby maximize the revival fitness), essentially all spores gave rise to a microcolony and thus the wt dominated (Fig. S7d, e).

We thus conclude that, either life-cycle strategy can outcompete the other, depending on the upshift condition.

### Closely related natural *B. subtilis* isolates have adopted different life-cycle strategies

In nature, closely related strains could have adopted different life-cycle strategies that are advantageous for survival in ecological niches that favor either quality or quantity. *B. subtilis* has traditionally been isolated from the soil but recently also from the feces of animals, including chickens, that ingest their food directly from the ground [7]. Recent natural isolates include PS216, which was obtained from a sandy riverbank in Slovenia [46, 47] and BSP1, which is a gut isolate from a broiler raised organically on an English farm [7]. The genomes of both strains have been sequenced [47, 48]. They are closely related to the laboratory strain B168 and are classified as *B. subtilis* subsp. *subtilis*. They each contain a single chromosome, are naturally competent and are thus genetically tractable [11, 49]. These two strains are therefore ideally suited to investigate the life-cycle strategies of closely related natural isolates.

We first checked whether natural isolates encounter a quality–quantity tradeoff (see Supplementary methods). A necessary condition for the emergence of a tradeoff is that spore quality is a function of sporulation timing (i.e., the time at which a spore is produced relative to the onset of starvation) regardless of spore age (i.e., the time that elapses between sporulation and spore revival). We utilized the fact that in isogenic populations some cells sporulate earlier than others [45, 50–52] and thus early spores should be of higher quality and revive better than later spores. To test this, we labeled spores with a fluorescent reporter (P_*rapA*_-mCherry) that distinguishes between early- and late-forming spores in a shaken culture [30]. Indeed, in both strains the revival frequency of early spores was higher than of the late spores (Fig. S8a, b) and these differences persist in both fresh and aged spore samples (Fig. S9). Interestingly, the gut isolate BSP1 produced a higher proportion of very early (i.e., top-quality) spores than the soil isolate PS216 and overall, BSP1 revived better than PS216 (Fig. S8c).

**Fig. 2:**
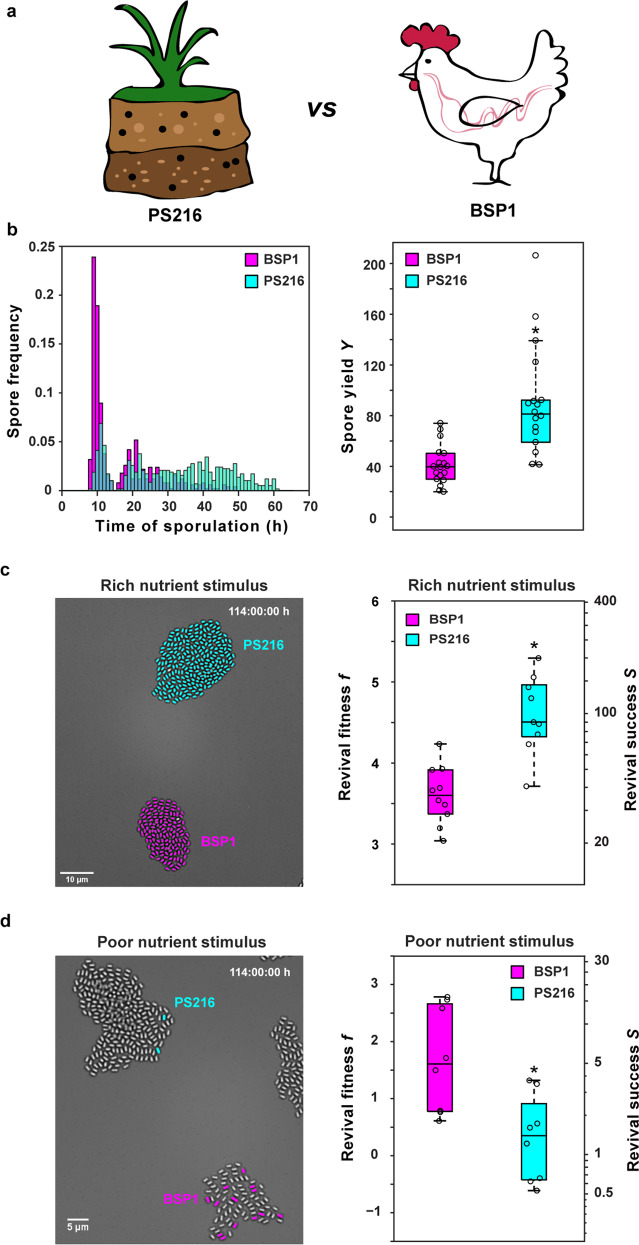
Natural *B. subtilis* isolates utilize different life-cycle strategies. **a** *Bacillus subtilis* strain PS216 is a soil isolate, while strain BSP1 was isolated from the broiler chicken gut. PS216 cells express mCherry to distinguish them from BSP1 cells. The two strains were competed against each other under fluctuating nutrient conditions using the microscopic life-cycle assay. See Movies S5 (10.5446/42830) and S6 (10.5446/42831). **b** Starvation response of BSP1 (magenta) and PS216 (cyan). Left: spore frequency distributions as a function of starvation time. Right: box-plot of the spore yield, defined as the number of spores generated per cell entering the nutrient downshift. The difference is significant based on a two-tailed Mann–Whitney *U* test. *U* = 23 (*U*_P=0.01_ = 75). **c** Spore revival in response to a rich nutrient stimulus (33% LB, 33% AGFK, and 33 mM l-alanine). Left: micrograph of spore microcolonies of PS216 and BSP1. Reviving spores are false colored. Right: box-plot of revival success, defined as the number of outgrowing spores per standard initial cell entering the nutrient downshift. The difference is significant based on a two-tailed Mann–Whitney *U* test. *U* = 6 (*U*_P=0.01_ = 7). **d** Spore revival in response to a poor nutrient stimulus (100 mM l-alanine). Left: micrograph of spore microcolonies of PS216 and BSP1. Reviving spores are false colored. Right: box-plot of the revival success. The difference is significant based on a two-tailed Mann–Whitney *U* test. *U* = 4 (*U*_P=0.01_ = 13). For all box-plots: *N* ≥ 8 colonies from *n* ≥ 2 technical replicates per condition.

Next, the soil isolate PS216 (labeled with P_*rapA*_-mCherry) was forced to compete with the gut isolate BSP1 in the microscopic life-cycle assay (Fig. 2a, Supplementary Movies S3 and S4). The two strains were mixed in a 1:1 ratio after growth in CH medium and their sporulation response was observed by microscopy as before. The initial response of the two natural isolates on SM* was comparable to that of the laboratory strain B168: the cells divided (sometimes after a lag phase of 1–2 h) and then grew into microcolonies. About 8 h later the first prespores appeared within the microcolonies. Although the onset of sporulation occurred at approximately the same time in both strains, the sporulation response was clearly distinct (Fig. 2b, left). BSP1 produced a large fraction of spores within a first wave of sporulation, and the majority of spores were formed during the first 30 h of starvation. On average, each BSP1 cell generated about 40 spores during the starvation period, again with substantial variation from colony to colony (Fig. 2b, right). By contrast, few PS216 cells sporulated during the first wave; most cells delayed sporulation, continuing to grow and divide. In these microcolonies, sporulation continued for several days, and a sizeable fraction of spores formed between 30 and 60 h after the imposition of starvation (Fig. 2b, left). As a result, each PS216 cell gave rise to about twice as many spores on average.

Together these data show that the life-cycle of natural isolates is subject to a quality–quantity tradeoff: the sporulation dynamics affect both the spore yield and the distribution of spore quality. While PS216 produces more spores than BSP1 by delaying sporulation in a subset of cells, BSP1 generates more high-quality spores than PS216. This indicates that the soil isolate PS216 is a spore-yield strategist, while the gut isolate BSP1 is a spore-quality strategist. If so, PS216 should be fitter than BSP1 under nutrient-rich upshift conditions, when most spores are able to revive. Conversely, when given a poor nutrient stimulus that stringently selects for high-quality spores, the quality strategist BSP1 should prevail. This was indeed the case (Fig. 2c, d). In response to a very rich nutrient upshift (LB plus a mixture of the germinant l-alanine and the germinant mixture AGFK to boost germination [24, 25] and thereby maximize the revival fitness), almost all spores of both BSP1 and PS216 were able to grow out successfully (Fig. 2c, left, Movie S5, 10.5446/42830). As a result, the revival fitness is dominated by the spore yield and PS216 generates about twice as many reviving spores as BSP1, resulting in a fitness advantage for PS216 over BSP1 (Fig. 2c, right). However, when revival was induced by supplying l-alanine, the revival fitness dropped sharply and only a small subset of spores from each strain succeeded in outgrowth (Fig. 2d, left, Movie S6, 10.5446/42831). As predicted, BSP1 produced more high-quality spores that were able to grow out than PS216 did. Thus, under poor nutrient upshift, BSP1 is fitter than PS216, despite its lower spore yield (Fig. 2d, right). We conclude that closely related isolates of *B. subtilis* have adopted different life-cycle strategies, and that either strategy can be beneficial for revival fitness, depending on the nutrient supply available during spore revival.

### *rap-phr* systems can confer adaptability to diverse revival conditions

**Fig. 3:**
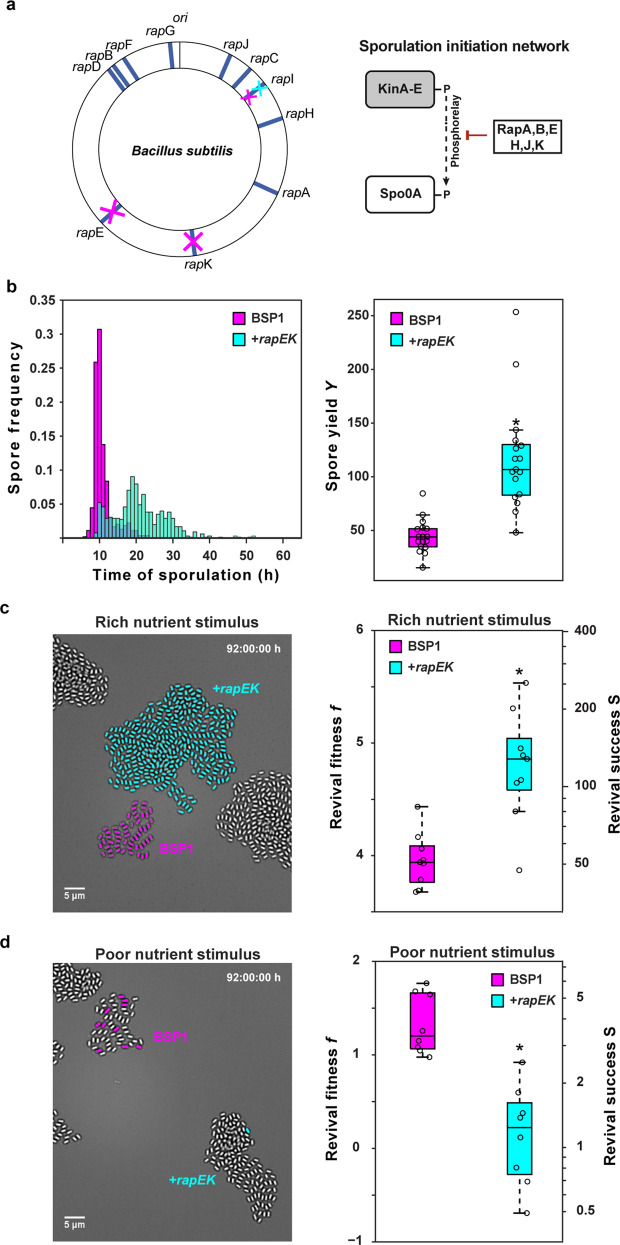
The quality strategist BSP1 can be converted into a PS216-like yield strategist by genetically modifying its sporulation network. **a** The genomes of BSP1 and PS216 differ with respect to the presence of *rap-phr* signaling systems that inhibit the onset of sporulation in the laboratory strain B168. Left: schematic map of *rap-phr* systems in the *B. subtilis* B168 genome. The *rap-phr* genes that are missing in BSP1 (magenta) and PS216 (cyan) are crossed out. Right: schematic depiction of the sporulation initiation network. **b** Starvation response of BSP1 (magenta) and a *rapEK+* strain (cyan) in a co-culture life-cycle assay. BSP1 cells express *cfp* to distinguish them from the *rapEK+* (short for *rapE-phrE rapK-phrK*) cells. See Movies S7 (10.5446/42832) and S8 (10.5446/42833). Left: spore frequency distributions as a function of starvation time. Right: box-plot of the spore yield (defined as the number of spores generated per cell entering the nutrient downshift). The difference is significant based on a two-tailed Mann–Whitney *U* test. *U* = 10 (*U*_P=0.01_ = 70). **c** Spore revival in response to a rich nutrient stimulus (33% LB, 33% AGFK, and 33 mM l-alanine). Left: micrograph of spore microcolonies of BSP1 and *rapEK*+. Reviving spores are false colored. Right: box-plot of revival success. The difference is significant based on a two-tailed Mann–Whitney *U* test. *U* = 0 (*U*_P=0.01_ = 7). **d** Spore revival in response to a poor nutrient stimulus (100 mM l-alanine). Left: micrograph of spore microcolonies of PS216 and BSP1. Reviving spores are false colored. Right: box-plot of revival success. The difference is significant based on a two-tailed Mann–Whitney *U* test. *U* = 7 (*U*_P=0.01_ = 11). For all box-plots *N* ≥ 8 colonies per condition from *n* ≥ 2 technical replicates.

The strains BSP1 and PS216 are genetically closely related both to each other and to the laboratory strain B168. They have a smaller genome than the laboratory strain, due mainly to the absence of prophages and mobile genetic elements in the chromosome [47, 48]: both strains lack the intergenic conjugative element ICEBs1 and the functional SPβ prophage, and BSP1 also lacks the SKIN element and prophage-like regions 3–6. Notably, these regions contain genes coding for regulators of sporulation induction, including the *rapI-phrI, rapE-phrE*, and *rapK-phrK* regulatory systems. Compared to the B168 laboratory strain, PS216 lacks *rapI-phrI*, while BSP1 lacks *rapI-phrI*, *rapE-phrE*, and *rapK-phrK* (Fig. 3a, left). Many *rap* genes code for phosphatases that promote the dephosphorylation of Spo0F [21–23], the first response regulator of the sporulation phosphorelay, which regulates the initiation of sporulation [19]. They thereby inhibit the flux of phosphoryl groups to Spo0A [21], the master regulator of sporulation [20], and thus delay the induction of sporulation (Fig. 3a, right). In particular, RapE [22] and RapI [23] are Spo0F phosphatases, and RapK has also been implicated in the inhibition of sporulation [11]. The activity of the Rap proteins is counteracted by their cognate Phr signaling peptides. This suggests that the differential representation of *rap-phr* systems in the genomes of BSP1 and PS216 could be responsible for their different life-cycle strategies. If so, introducing the *rapE-phrE* and *rapK-phrK* signaling systems into the BSP1 genome should cause this strain to abandon the quality strategy in favor of a PS216-like yield strategy.

To test this hypothesis, we competed BSP1 with *a rapEK*+ transplanted strain (short for BSP1 *rapE-phrE rapK-phrK*), into which both regulatory systems had been introduced at an ectopic locus in the chromosome [11]. As expected, the sporulation frequency distribution of the *rapEK*+ strain was shifted to longer delay times with respect to BSP1 (Fig. 3b, left), although whether this occurs via a change in the efficiency of phosphotransfer to Spo0A remains to be verified. While the majority of BSP1 spores appeared during a single wave of sporulation events after around 10 h of starvation, only a small subset of spores of the *rapEK*+ strain was generated during this time. Instead most of its spores formed in a second wave of sporulation events that peaked around 20 h of starvation and persisted for up to 40 h. Indeed, occasional late spores were formed even after 50 h of starvation. The delay in sporulation seen in the *rapEK*+ strain was accompanied by additional cell divisions in the microcolony (Movies S7, 10.5446/42832 and S8, 10.5446/42833). As a result, the average spore yield more than doubled to more than 100 spores (Fig. 3b, right). Thus, the yield of the *rapEK*+ strain was indeed comparable to that of PS216, indicating that the additional *rap-phr* systems in the genome shift the life-cycle strategy toward the generation of higher spore yields. Moreover, in response to the rich nutrient stimulus, almost all spores revived successfully (Fig. 3c left, Supplementary Movie S7). As a result, the revival success of the *rapEK*+ strain, acting as the yield strategist, was more than twice as high than BSP1 (Fig. 3c, right). Conversely, in response l-alanine, revival success dropped (Fig. 3d left, Supplementary Movie S6) and the *rapEK*+ strain was less fit than BSP1 (Fig. 3d, right).

To summarize, the *rapE-phrE* and *rapK-phrK* systems have a profound effect on the life-cycle strategy of *B. subtilis*, in accordance with the quality–quantity tradeoff model. By delaying sporulation, they increase the overall spore yield, which is beneficial only under rich nutrient upshift conditions.

## Discussion

Our results challenge the traditional view of sporulation as an isolated stress-response trait [12, 13, 17, 18]; instead sporulation and spore revival jointly determine fitness in a fluctuating environment. Different spore-revival conditions favor alternative sporulation strategies that increase either spore yield or spore quality at the expense of the other trait, both in a synthetic model strain and in natural isolates. We therefore propose that selection for spore revival contributes to the evolutionary adaptation of sporulation traits and the sporulation initiation network in particular. This is exemplified by the variations in the numbers of *rap-phr* signaling systems found in different *B. subtilis* isolates that increase spore yield at the expense of spore quality. Notably, *rap-phr* signaling systems that delay sporulation are often found on phages, conjugative transposons and plasmids. Hence their positive effect on host fitness may serve to maintain their presence in cells, e.g., the functional *rapE* is part of a defective prophage [22]. Genetic adaptation could also affect other sporulation regulators, e.g., sporulation kinases [53], and it is likely that the same principle applies to other *Bacilli* [54–56].

In most organisms, the molecular basis of quality–quantity tradeoffs is not well understood and this also applies to sporulating bacteria. The enzyme Ald is one of presumably several quality determinants of spores in the *B. subtilis* laboratory strain. The enzyme promotes spore revival under challenging nutrient conditions in which metabolism of the nutrient–germinant l-alanine in the germinated spore appears to be decisive for successful outgrowth (Fig. S1). Whether Ald contributes to spore quality also in natural isolates remains to be verified. One hint that this might indeed be the case is provided by the fact that we detected fluorescence from an Ald-mCherry fusion protein in spores of both the chicken and the soil isolate; moreover, revival frequency was positively correlated with spore fluorescence (Fig. S10). Regardless of the molecular details, our experiments demonstrate that natural isolates encounter a spore quality–quantity tradeoff, which affects their fitness in different environments.

In higher organisms, tradeoffs play an important role in the evolution of life-history strategies during adaptations to different niches, and contribute to maintenance of biodiversity [57, 58]. Since spores disperse easily, obscuring life-history trajectories, it is generally very difficult to discern such relationships from studies of natural isolates [1]. *B. subtilis* spores are excreted from the chicken gut together with digested food within hours [59] and do not persist in the gut for longer than a few days [60]. However, due to coprophagy, the spores are presumably taken up again. Hence, on a farm, *B. subtilis* could take advantage of proliferation in the gut, in which case spores must revive quickly. Therefore, selection for spore quality might contribute to the fast sporulation kinetics of farm isolates [7].

Finally, the existence of a spore quality–quantity tradeoff implies that sporulation and spore-revival traits cannot evolve independently, which could contribute to the resistance of *B. subtilis* to attempts to evolve sporulation in the laboratory [12, 13]. Since sporulation and spore revival are important features of the *B. subtilis* life-cycle that contribute to its industrial application as a biocontrol agent and probiotic, respectively [15, 16], laboratory evolution experiments targeting the complete life-cycle could pave the way for the development of the next generation of spore-based probiotics.

In conclusion, an evolutionary and ecological understanding of sporulation requires consideration of the entire bacterial life cycle of sporulation and spore revival, and will benefit from the development of life-cycle theories in analogy to life-history theory in higher organisms [38, 39] to address how quality–quantity tradeoffs shape evolution across all taxa.

## Supplementary information

Supplementary Material
