# Supplementary material for: A spore quality–quantity tradeoff favors diverse sporulation strategies in Bacillus subtilis
Source: ISME J. 2020 Jul 28;14(11):2703–14. doi: 10.1038/s41396-020-0721-4 (PMC7784978; doi:10.1038/s41396-020-0721-4)
Supplement: Supplementary file 1 — Supplementary Material [file 41396_2020_721_MOESM1_ESM.pdf]

SUPPLEMENTARY MATERIAL

A spore quality-quantity tradeoff favors diverse sporulation strategies in *Bacillus subtilis*

Mutlu et al.

Includes Supplementary Text, Supplementary Methods, Supplementary Figures S1-S10, Supplementary Movies S1-S8, Supplementary Tables S1-S3 and Supplementary References

SUPPLEMENTARY TEXT

Knowledge of the molecular mechanisms that underlie the spore quality-quantity tradeoff is not required in order to investigate its effects on fitness. Nonetheless, insights into spore-quality determinants could help to identify conditions that might select for different sporulation strategies.

In a previous study [1] we provided evidence that the enzyme alanine dehydrogenase (Ald) affects spore quality. Here, we summarize previous findings and provide additional experimental evidence that Ald is a spore quality determinant. We then use this knowledge to develop a mechanistically inspired life cycle model that enables us to investigate theoretically under what conditions a spore quality-quantity tradeoff is likely to be of relevance to fitness, and use the model to guide the fitness experiments in this study.

1. Evidence for Ald as a determinant of *B. subtilis* spore quality

The sporulation strategy determines Ald levels in spores

For most, if not all, organisms the underlying molecular mechanisms that give rise to a quality-quantity tradeoff are still poorly understood. In higher organisms it is typically assumed that the tradeoff arises as a consequence of limited resources available for investment in reproduction. The limiting resource can be assigned to the generation of progeny in accordance with various resource allocation schemes that control the number and quality of offspring in varying proportions. In a recent study we provided evidence that the metabolic enzyme alanine dehydrogenase Ald may be a limiting resource in starving *B. subtilis* cells. Ald is distributed among the derived ("daughter") spores following a phase of pre-sporulation vegetative growth during which Ald levels are diluted with each cell division that occurs prior to sporulation. The sporulation strategy determines the length of the pre-sporulation growth phase, and therefore, the amount of Ald available for allocation to the derived spores [1].

We have previously studied the function of Ald during starvation, sporulation and spore revival in time-lapse microscopy experiments. We found that transcription of *ald* is strongly down-regulated, if not completely repressed, under starvation stress, presumably as a result of starvation for L-alanine. Consequently, the total amount of Ald stored in each cell upon entry into the starved state may be considered to be a fixed resource. With each subsequent cell division, Ald proteins will be shared among daughter cells and thus Ald levels should decay exponentially (assuming that the protein is stable). Upon sporulation, the cell divides asymmetrically and the smaller forespore compartment subsequently gives rise to the spore

core. Hence, the Ald concentration in the spore should correlate with Ald concentrations in the corresponding sporulating cell.

This model makes several testable predictions. First, if Ald is a fixed resource during starvation, Ald levels in the spore should decrease exponentially with sporulation timing. We tested this prediction by correlating spore fluorescence from an mCherry-labeled Ald protein with sporulation time by re-analyzing time-lapse movies of sporulating microcolonies acquired previously (see https://av.tib.eu/media/33997 for a representative movie). As expected, we find that spore fluorescence levels decay exponentially with sporulation time (Fig. S1a). That is, spores produced earlier in time have a higher Ald levels than spores produced later in time. Moreover, the timescale of fluorescence decay (0.14 h^{-1}) matches well with the cellular division rate (0.15 h^{-1}).

Another prediction of this model is that more rapid sporulation should shift the distribution of Ald concentrations in spores to higher levels, as fewer cell divisions occur prior to sporulation. We previously showed that the average spore fluorescence from Ald-mCherry increased when sporulation was accelerated by induction of a sporulation kinase KinA driven by an IPTG-inducible promoter. To further substantiate our previous finding, we determined the corresponding fluorescence distribution as a function of IPTG levels. The histograms clearly show the predicted shift in the distribution to higher fluorescence intensities (Fig. S1b). We next repeated this experiment in a liquid sporulation culture and again observed the shift of the fluorescence distribution to higher levels with increasing IPTG levels (Fig. S1c). Together, these data confirm that the levels of Ald in spores are controlled by the sporulation strategy on both solid and in liquid media.

Ald is a spore quality determinant

Since sporulation timing controls both the number of spores produced from a single progenitor cell and the corresponding distribution of Ald levels in the spores, a quality-quantity tradeoff emerges – provided that Ald controls ‘spore quality’. The proposition that Ald is a spore quality determinant is supported by several experimental findings [1]: 1) the spore revival frequency upon adding the nutrient-germinant L-alanine to spores on agarose pads containing (exhausted) sporulation media is correlated with Ald levels in the spores, 2) spores of an Δ*ald* strain germinated normally but were blocked in spore outgrowth under the same experimental conditions, and 3) the spore outgrowth frequency could be increased beyond wt levels by overexpressing Ald in progenitor cells.

Ald is an enzyme that metabolizes L-alanine to pyruvate, and has been implicated in metabolization of the nutrient germinant L-alanine in germinated spores [2]. We thus hypothesized that the enzyme plays a role in bootstrapping metabolism in the reviving spore. We tested this by monitoring the onset of metabolic activity in spore suspensions with the help of a colorimetric assay using the redox dye Alamar Blue (resazurin) [3, 4]. The dye is reduced as germinated spores regain their metabolic activity (and replenish their NADH and NADPH pools[5]), shifting its absorption maximum from 605 nm to 575 nm. This color change can be detected by a photospectrometer by measuring the ratio $R = A_{575\ \mathrm{nm}}/ A_{605\ \mathrm{nm}}$ of absorbance A (or optical density (OD)) at 575 nm and 605 nm, respectively of a germinating spore suspension (Fig. S1d, see Supplementary Methods for details). For Δ*ald* spores the color change indeed occurred at a slower rate than for wt when induced with L-alanine (Fig. S1d, left panel). For stimulation with rich media (LB plus nutrient germinants L-alanine and AGFK) the color change occurred more rapidly but the rates were comparable in both strains (Fig. 1d, right panel). No response occurred when water was added, which excludes unspecific conversion of the dye (Fig. S1d, dotted lines) while revival without a dye increased R to a higher but constant value (Fig. 1d, Inset). Since the overall color change is affected by the metabolic activity of germinating spores and their numbers, we confirmed that Δ*ald* spores germinate like wt [1] on L-alanine by measuring the changes in optical density (Fig. S1e, left panel). We thus conclude that Δ*ald* spores show a lower metabolic activity compared to wt when L-alanine is the sole external nutrient source. In rich media other nutrients may compensate for this metabolic deficiency, as wt and Δ*ald* then show similar rates of color change (Fig. S1d, left panel) at an overall comparable revival kinetics, perhaps even with a slight kinetic advantage of Δ*ald* over wt spores (Fig. S1e, right panel).

Together these data further support the idea that Ald acts as a spore quality determinant, which affects the capacity of spores to metabolize the nutrient germinant L-alanine. Notably, in rich nutrient media, other pathways contribute to the metabolism of the germinated spore. As a result, the Ald pathway may become dispensable for outgrowth.

2. Mathematical life cycle model for sporulation and revival

Sporulation. We describe the kinetics of sporulation by a minimal model introduced in Ref. [1] and depicted in Fig. S2a. At time $t = 0$, a single bacterium in a naïve vegetative state c_0 is placed in nutrient-depleted sporulation medium. As the seed bacterium and its progeny progressively consume the available nutrients and/or intracellular reserves, they transition to starvation. This process is modelled as a passage through a series of vegetative states $c_1, \ldots, c_k$,

with transition rate η. In all vegetative states bacteria grow with unchanged rate λ; however, in the final starved state c_k, cell death μ occurs at a rate μ that exceeds the growth rate, $\mu > \lambda$. From all non-naïve vegetative states $c_1, \ldots, c_k$, bacteria can also differentiate into spores at a constant rate δ. We note here that sporulation from stages $i < k$ is necessary to reproduce a broad distribution of spore-formation times, as generally observed in our experiments. We denote the number of cells in state i at time t by $c_i(t)$.

In response to starvation at $t = 0$, bacteria switch off production of enzymes required for bootstrapping metabolism upon revival; this was shown to be the case for alanine dehydrogenase (Ald) in Ref. [1] with additional evidence provided in Fig. S1, but other such quality-marker enzymes are likely to exist. For concreteness we focus on Ald here. As cells continue to grow, the Ald amount a in each cell decreases as $a(t) = a_0 e^{-\lambda t}$ (see Fig. S1a), while the total amount of Ald in the clone remains constant (at a_0). At the moment t when a spore is produced, the amount of Ald present in the cell, $a(t)$, is encapsulated into the spore, which thereby acquires the spore quality $q = a(t)$. We denote the number of spores with quality q at time t by $s^q(t)$.

The resulting kinetic equations are:

$$\partial_t c_0(t) = (\lambda - \eta) c_0(t) \quad \text{Eq.(1)}$$

$$\partial_t c_i(t) = \eta c_{i-1}(t) + (\lambda - \eta - \delta) c_i(t) \;\text{ for }\; 0 < i < k \quad \text{Eq.(2)}$$

$$\partial_t c_k(t) = \eta c_{k-1}(t) + (\lambda - \mu - \delta) c_k(t) \quad \text{Eq.(3)}$$

$$\partial_t s^q(t) = \delta_D[q - a(t)] \delta \sum_{i=1}^{k} c_i(t) \quad \text{Eq.(4)}$$

Where δ_D denotes the Dirac delta function. Eq. 4 thus indicates that $s^q(t)$ increases in a step-like manner at the time when $a(t) = q$. The system of linear equations Eq. 1 to Eqs. 4 is readily solved numerically or symbolically for the relevant initial conditions: $c_0(0) = 1$ and all other populations zero at $t = 0$.

Spore-quality density and yield. The process of starvation and sporulation produces spores of variable quality. We define the spore-quality density $\sigma(q)$ as the number density of spores generated with quality q. The spore-quality density $\sigma(q)$ can be determined from the solution of Eqs. (1) –(4). We obtain:

$$\sigma(q) = \int_0^\infty \partial_t s^q(t)dt = \frac{\delta}{\lambda q}\sum_{i=1}^{k} c_i\left(\lambda^{-1}\ln\left[\frac{a_0}{q}\right]\right) \qquad \text{Eq.(5)}$$

The number of spores within a given quality range is then obtained by integrating over $\sigma(q)$. The total spore yield (per initial cell) is obtained by summing the spore quality density over all qualities as

$$Y = \int_0^\infty \sigma(q)dq = \delta\sum_{i=1}^{k}\int_{t=0}^{\infty} c_i(t)dt. \qquad \text{Eq.(6)}$$

Revival. The probability of a spore of quality q to revive in an outgrowth medium with nutrient level m is denoted $P_m(q)$. The product $P_m(q)\sigma(q)$ then defines the density of revived spores. Thus, the average total average number of revived spores per initial cell is given by:

$$S_m = \int_0^\infty \sigma(q)P_m(q)dq. \qquad \text{Eq.(7)}$$

and determines the revival success S_m in a given revival environment.

The revival fitness is then defined as the natural logarithm of the revival success

$$f_m = \ln(S_m). \qquad \text{Eq.(8)}$$

It provides a measure for the relative increase or decrease of the population size per starvation-revival cycle and is therefore analogous to the logarithmic growth rate, which measures relative changes in population size per time. In essence, revival fitness is determined by the fraction of spores that are of sufficient quality to grow out in the given outgrowth medium. This fraction depends on both the sporulation process (via σ) and on the revival process in the given environment (via P_m). In this way, the minimal life-cycle model presented here links sporulation kinetics and revival fitness of spores.

Quality-outgrowth relation. In the absence of direct measurements, we resort to describing the quality-outgrowth relationship $P_m(q)$ by a simple model that is motivated by the basic physiology of spore revival (Fig.S3a). We assume that revival is gated by a bottleneck in an overall metabolic flux j required within the germinated spore to bootstrap vegetative metabolism. In other words, the spore will regrow with high probability if the metabolic flux j

exceeds some universal, medium-independent threshold value j_*, but for decreasing j, its chance of reviving progressively decreases.

The quality marker − the enzyme alanine dehydrogenase Ald − metabolizes L-alanine in germinated spores [2] and is required for alanine-dependent outgrowth [1], but is required in much lower amounts or even not at all in rich medium [1] (Fig. S1d, e). We tentatively attribute this fact to the presence of alternative nutrient sources in the medium, made available through alternative enzymatic pathways, which also contribute to the metabolic flux j. The total flux is then composed of an alanine-dependent, Ald-mediated and therefore spore quality-dependent flux j_{Ala} and an alanine-independent, alternative flux j_{alt} maintained by other nutrients present in concentration m in the regrowth medium. That is,

$$j(q,m) = j_{\mathrm{Ala}}(q) + j_{\mathrm{alt}}(m) \qquad \text{Eq.(9)}$$

In the following, for simplicity, we consider the alanine content in all outgrowth media to be equal and non-limiting, so that

$$j_{\mathrm{Ala}}(q) = \alpha q \qquad \text{Eq.(10)}$$

is proportional to the spore quality alone. Here, the proportionality constant α is the flux per unit amount of Ald, *i.e.* the Ald efficiency. By contrast, the alternative flux j_{alt} strongly depends on the chosen outgrowth medium; it is a measure of the richness of the medium. We take j_{alt} to be independent of spore quality, which amounts to the model assumption that the alternative enzymes carrying the alternative flux are not subject to dilution. This assumption is valid if their production continues during starvation, or if these enzymes are diluted during starvation but always remain at high enough concentration to be non-limiting for j_{alt}.

To complete the model, we take the outgrowth probability to be a saturating function of the total flux. Specifically,

$$P_m(q) = \frac{j(q,m)}{j(q,m) + j_*} = \frac{\alpha q + j_{\mathrm{alt}}(m)}{\alpha q + j_{\mathrm{alt}}(m) + j_*} = \frac{q + j_{\mathrm{alt}}(m)/\alpha}{q + j_{\mathrm{alt}}(m)/\alpha + j_*/\alpha}, \qquad \text{Eq.(11)}$$

where j_* sets the typical flux needed for revival. Note that within this complementary flux model, any spore can grow out if the medium is rich enough, i.e. when $j_{\mathrm{alt}} > j_*$. Because the Ald efficiency α acts as a scaling factor for the fluxes, the outgrowth probability only depends on the rescaled fluxes j/α and thus Eq.(11) can be rewritten as:

$$P_m(q) = \frac{j(q,m)}{j(q,m) + j_*} = \frac{q + q_m}{q + q_m + q_*} \qquad \text{Eq.(12)}$$

Here, $q_* = j_*/\alpha$ defines an apparent spore quality threshold, which is the effective spore quality required for reviving with 50% probability on poor media by utilizing alanine; $q_m = j_{\text{alt}}(m)/\alpha$ is the apparent gain in quality that a rich medium supplies to the spore, compared to alanine only. This implies that when $q_m \gg q_*$ (or equivalently $j_m \gg j_*$) in very rich media, revival is dominated by the metabolic flux provided by the alternative nutrients and thus any spore will revive, regardless of its quality.

Model Parameters

Introduction of non-dimensional parameters (parameter reduction)

The dynamic equation Eq.(1) to Eq.(4) are non-dimensionalized by measuring time t in units of $\tau = 1/\lambda$, which amounts to setting $\lambda = 1$ in all equations. The kinetic rates η, μ and δ are now dimensionless and expressed relative to the growth rate λ. The parameter a_0 sets the overall scale for quality. The maximal quality $q_{\text{max}} = a_0$ is achieved when sporulation is immediate. To remove this irrelevant scale, we set $a_0 = 1$ in all equations. In effect, we measure quality (including q_* and q_m) relative to the maximal quality q_{max}.

Parameter values

For our minimal model, we consider a sporulation model with $k = 3$ non-naïve vegetative states and a starvation rate of $\eta = 1.3$, which indicates that on average, $3/(1.3 \times \ln 2) = 3.3$ doubling times are needed to progress to the fully starved state c_3. In the fully starved state, death is $\mu = 1.5$ times more likely than another division.

To model different life cycle strategies we vary the sporulation rate δ. To give a specific example, for fast sporulation with $\delta = 3$, sporulation happens three times faster than replication, while for slow sporulation with $\delta = 0.25$, sporulation is four times slower than replication. Fig. S2b shows the corresponding spore-quality densities resulting in a quality strategy (few spores of high quality) and a yield strategy (more spores but of lower quality), respectively.

To enable spore quality to be limiting for spore revival, we take $q_* = 1$, that is, the apparent quality threshold for revival matches the maximal spore quality.

To model different revival environments we vary the apparent gain in quality q_m that is supplied by increasing the "richness" of the revival medium. Specifically, $q_m = 0$ is a nutrient-poor environment, which thus selects for high quality spores. In contrast, in very nutrient-rich environment $q_m \gg 1$, any spore revives, regardless of its quality. Fig. S3b shows the resulting density profiles of revived spores *vs.* spore quality for the two life cycle strategies described in a poor environment $q_m = 0$ and a moderately rich environment with $q_m = 1$, respectively.

Effects of the spore quality-quantity tradeoff on revival fitness

Fig. S4 shows the revival fitness f (and the revival success S) as a function of the sporulation rate δ for different environments. Clearly, when all spores are able to revive regardless of their intrinsic spore quality due to the richness of the medium ($q_m \gg q_*$), a yield-optimizing strategy will optimize the revival fitness. Thus, in very rich media, revival success equals yield, $S \rightarrow Y$, so that slow sporulation becomes optimal. (In passing we remark that the optimal sporulation rate never approaches 0 in rich media, since in this limit, cells will die of starvation non-productively, reducing Y at very low δ.) Conversely, in a poor medium where $q_m \simeq 0$, only spores of sufficiently high quality q have a good chance of revival, and a quality strategy may pay off. Whether the increased quality over-compensates for the decrease in yield depends on the details of the spore-quality profile and revival probability.

As Fig. S4 shows, a swap of optimal strategies indeed occurs in the present model: While the fitness decreases in a nutrient-poor environment for any sporulation strategy, the decrease is minimized when spore quality is optimized. The dashed line in Fig. S4 shows how the optimal sporulation shifts from slow sporulation to fast sporulation as the richness of the medium decreases. In effect, the optimal sporulation rate is slow (corresponding to a yield strategy) for rich media and fast (quality strategy) for poor media. Interestingly, in an intermediate range of medium richness, the choice of sporulation strategy has almost no bearing on fitness.

SUPPLEMENTARY METHODS

Strains and plasmids

Plasmid construction

pSac-Kan_P*rapA*II-mCherry (EIB492): The T*gyrA* P*rapA*II-*mCherry* fragment was excised from EIB432 by restriction digestion with EcoRI, and inserted into the similarly cleaved vector pSac-Kan [6]. The plasmid was sequenced with primers DW119 and ST243.

Strain construction

The Δ*ald* knockout strains (BIB1396 and BIB1397) were constructed via transformation of BIB1392 with a *ald::mls*^{R} fusion product. *ald*-up, *ald*-down fragments were amplified from the *B. subtilis* 168 1A700 genome with MA51/MA52 and MA53/MA54 primer pairs, respectively. The *mls*^{R} cassette was amplified from pDG647 [7] with primers DW34 and DW35. The resulting fragments were fused by overlap-extension PCR using the end primers MA51 and MA54. Transformants were checked via colony PCR with the primers KN1, KN2, KN3 and LA61.

Colorimetric measurement of metabolic activity (Alamar Blue Assay)

The Alamar Blue assay was adapted from (Nagler, 2014) [4]. The titer of purified spores was assessed by microscopy and spore concentration was adjusted to $5*10^8$ spores ml^{-1}. Spores were heat inactivated for 10 minutes at 80°C and washed twice with MiliQ water. Then 100-µl samples of spore suspension were mixed with 80-µl aliquots of Assay Buffer (62.5 mM Tris HCl pH 7, 0.025% Tween20, 0.25 mg/ml Alamar Blue) in a 96-well microtiter plate. Spore suspension were incubated at 37°C with linear shaking at 1096 cpm (1 mm throw) in an EPOCH2 microplate reader. Revival was induced by adding 20 µl of a 1 M L-alanine solution or LB-Mix (33% LB, 33% AGFK, 33 mM L-alanine), respectively. To monitor the color change we measured absorbance (A) at 605 nm (A_{605nm} blue) and 575 nm (A_{575nm}, pink) at regular intervals over 3 hours. The absorbance signal A (*t*) was determined by averaging results from three technical replicates. The ratio $R = A_{575nm} / A_{605nm}$ was then used as a measure for reporting on the color change. A control without the dye was performed for each sample to monitor for changes in optical density $OD_{605\ nm}$ due to spore refractivity loss upon germination, and outgrowth.

Fluorescence-based spore analysis

To determine spore fluorescence levels, spores were segmented from bright-field images using Fiji software or a custom-written Matlab program, respectively. The fluorescence intensity was determined by subtracting the background fluorescence. Where applicable, individual spores were classified into groups according to specific criteria as described in the text (e.g. higher or lower intensity than median fluorescence of the spore population).

Fitness assays using spore mixtures produced in well-mixed liquid sporulation medium

Sporulation: Cells grown overnight in selective CH medium were re-inoculated in fresh CH medium (without selection) and grown to an OD_{600nm} of 0.8 – 1.0. Cells were then re-suspended in SM^A medium (SM medium + 1 mM L-alanine) (final OD_{600nm} = 0.5). Wells of a FlowerPlate (m2p-labs) were each filled with 1.2 ml of the cell suspension. Where indicated, IPTG was added to the wells. The culture was incubated for 4 days at 37°C with rigorous shaking (900 rpm) and air humidity was maintained at 75% in an Infors Multitron Pro 3mm micro-titer plate shaker. Spores were harvested and washed twice with MiliQ water and stored at 4°C before being assayed for revival.

Revival fitness: For revival assays, 4 µl spore suspension was spotted on agarose pads containing SM* media without L-alanine supplementation. These modified pads do not support spore revival without addition of further nutrients. Revival was induced by adding 10 µl of either 100 µM L-alanine solution or LB-germinant mix (33% LB, 33% AGFK, 33 mM L-alanine) on top of the pads, and the response was monitored by time-lapse microscopy. Since outgrowing spores will initiate the formation of microcolonies, the number of (micro)colony forming units (mCFU) was approximated by manually counting the number of outgrowing spores in each movie.

Sporulation of natural isolates in well mixed cultures

For the initial set of experiments (Fig. S8), spores were sporulated in culture tubes. Cells grown overnight in selective CH medium were re-inoculated in fresh CH medium (without selection) to grow to an OD_{600nm} of 0.8 – 1.0. Cells were then re-suspended in SM medium (final OD_{600nm} = 0.5) and incubated for 4 days in a culture tube with shaking at 37 °C. Next, the spores were washed twice with phosphate-buffered saline (PBS) and stored at room temperature until being assayed for revival.

For all other experiments (Fig. S8 & Fig. S9) spores were produced in microtiter plates using a micro-plate shaker, as described in the fitness experiments. Spores were harvested after 4 days, if not otherwise indicated. Spores were washed twice with MiliQ water directly after harvesting. Spores of the natural isolates (strains BIB1249, BIB1282, BIB2115 and BIB2117) were heat inactivated at 80°C for 10 minutes to kill any residual vegetative cells and were washed twice with MiliQ water immediately before the revival assay.

SUPPLEMENTARY FIGURES

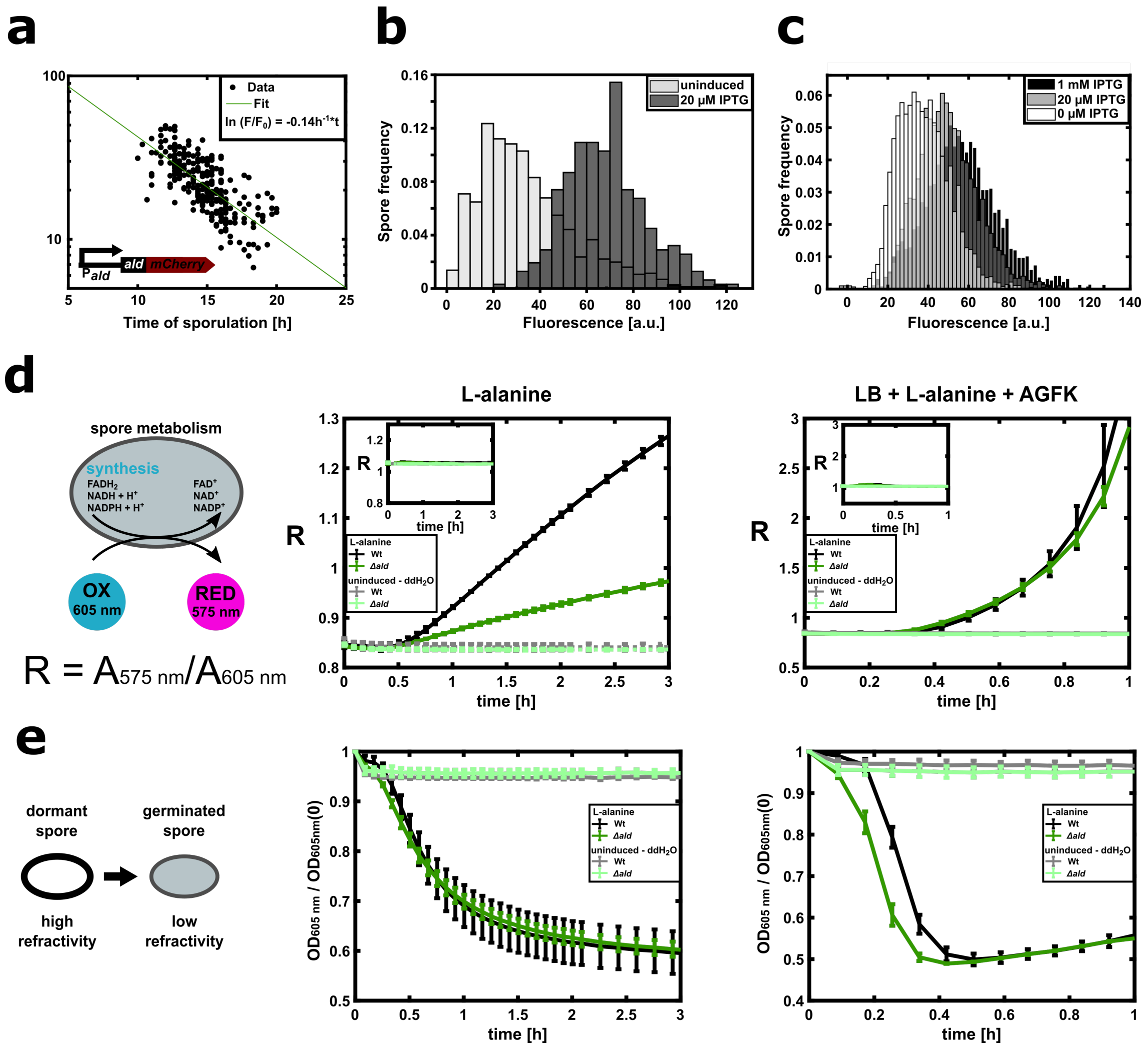

Figure S1. Alanine dehydrogenase (Ald) is a spore quality determinant. (a) Spores contain Ald and levels decrease with sporulation timing. Results from re-analysis of a time-lapse experiment following sporulation of the P_{ald}-Ald-mCherry reporter strain to give rise to heterochronously sporulating microcolonies. Spore fluorescence decreases exponentially as a function of sporulation time. The decay rate is 0.14 h^{-1}, which matches well with the cellular division rate (0.15 h^{-1}). **(b, c)** The Ald spore fluorescence distributions shift to higher levels when sporulation is accelerated by inducing expression of the sporulation kinase gene *kinA* from an IPTG-inducible promoter in spores produced on agarose pads (**b**) and in liquid sporulation medium (**c**). **(d)** Results of an Alamar Blue assay of wt (black) and *Δald* spores (green). The dye Alamar blue is reduced by redox active compounds produced as germinating spores initiate metabolism, which shifts its absorption maximum from 605 nm to 575 nm. The ratio $R = A_{575\ nm}/A_{605\ nm}$ of absorbance A at 605 nm and 575 nm, respectively is plotted as a function of time after induction of the spore suspension with L-alanine (left panel) or LB-Mix (33% LB, 33% AGFK, 33 mM L-alanine) (right panel). As a control for unspecific reduction of the dye, spores were induced with water (dotted lines). The Inset show results from negative controls without dye. Error bars

show the standard deviation of two biological replicates (based on averaging three technical replicates each). Spore suspensions of a *Δald* strain induced with L-alanine reduce the dye at a slower rate than wt, indicating a lower metabolic activity. See Supplementary Text and Supplementary Methods for details. **(e)** Germination decreases the refactive index of spores which decreases the optical density (OD) of a spore suspension upon germination. The change in $OD_{605\ nm}$ relative to the initial value before induction is plotted as a function of time after adding L-alanine (left panel) or LB-Mix (right panel) for wt (black) and *Δald* spores (green). Dotted lines show a control, to which only H_20 was added. Error bars show the standard deviation of two biological replicates (based on averaging three technical replicates each).

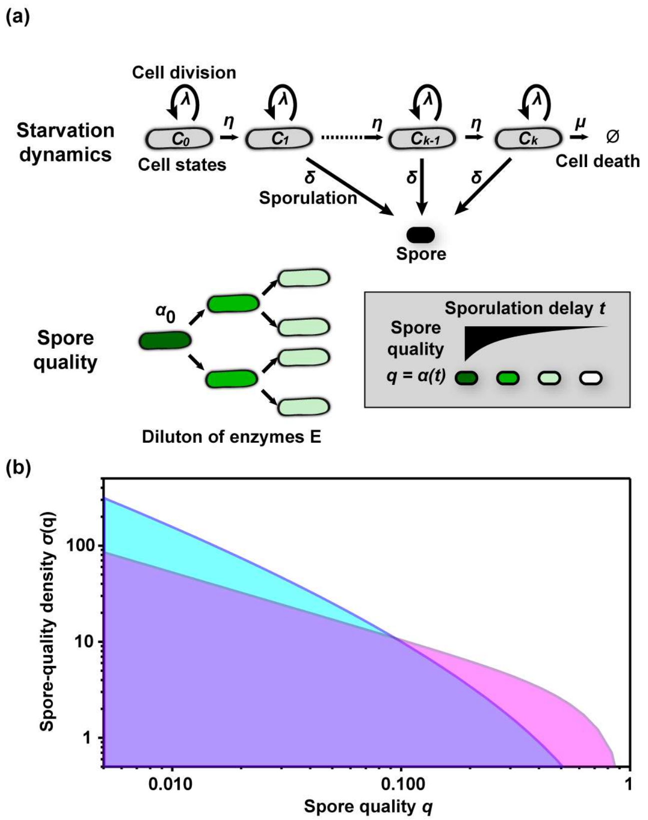

Figure S2. Spore-quality model. (a) Sporulation model. Top: Vegetative cells progress towards starvation, continuing to grow and divide, and forming spores along the way, with rates η, λ and δ, respectively. Starved cells die with rate μ. We introduce dimensionless rates by measuring time in units of the characteristic cell division time $\tau = 1/\lambda$. Then all rates become dimensionless, i.e. $\lambda = 1$ and all other rates are now expressed relative to λ. Bottom: As sporulation is delayed by time t, the quality marker a is diluted from its initial value a_0 by cell growth with rate λ (left). Upon sporulation, the enzyme is stored in the spore. Thus, the spore quality q decreases exponentially with increasing delay time (right). The parameter a_0 sets the overall scales for quality and denotes the maximal spore quality $q_{\max} = a_0$ that can be achieved upon immediate sporulation. We thus normalize all quality related quantities q to $q_{\max} = a_0$. **(b)** Spore-quality density $\sigma(q)$, defined as the number density of generated spores with quality q resulting from the sporulation model with $k = 3$ non-naïve vegetative states, at fast and slow sporulation rates δ (magenta and cyan, respectively). Quality q is expressed relative to a_0 and thus $q_{\max} = 1$. Dimensionless rate parameters are expressed relative to the growth rate λ, i.e. $\lambda = 1, \eta = 1.3, \mu = 1.5, \delta = \{3.0, 0.25\}$.

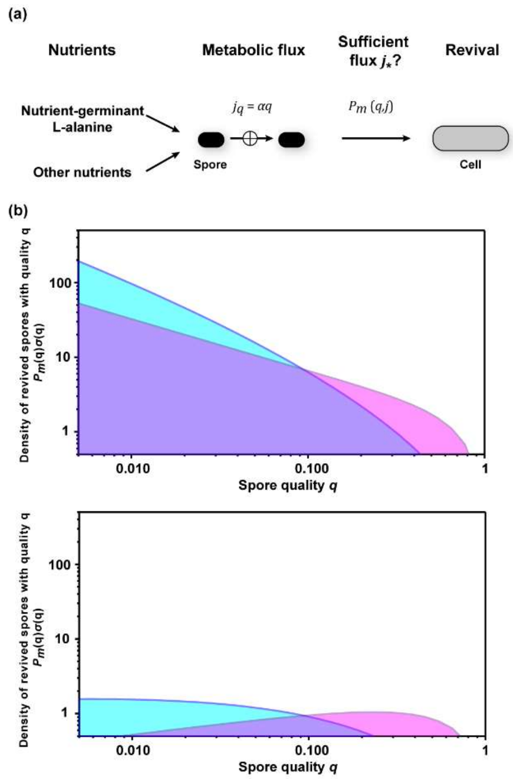

Figure S3. Spore-revival model. (a) The probability of successful spore revival $P_m(q)$ depends on the metabolic flux in the germinated spore, which powers spore outgrowth. The metabolic flux $j(q,m) = j_q + j_{alt}(m)$ comprises a quality-dependent flux and an alternative, quality-independent flux. The quality-dependent flux scales linearly with the level q of the spore-quality marker Ald, which is required to metabolize the nutrient-germinant L-alanine, i.e. $j_q = \alpha\, q$. The probability P for a spore to revive depends on j and thus on spore quality q. We introduce $q_* = j_*/\alpha$, which denotes an apparent spore quality threshold that is required for reviving with 50% probability by utilizing alanine only. The "richness" of the medium m supplied during nutrient upshift modulates the strength of the alternative flux by providing other substrates. In effect $q_m = j_{\text{alt}}(m)/\alpha$ is the apparent gain in quality that a rich medium supplies to the spore, compared to alanine only. **(b)** Number density $P_m(q)\sigma(q)$ of revived spores with quality q, for fast (magenta) and slow (cyan) sporulation rates assuming $q_* = 1$. Thus, only spores with an overall apparent quality close to $q_{\text{max}} = 1$ revive with high probability. Top panel: In rich medium ($q_m = 1$), the quality-independent flux dominates, and thus the density of revived spores mirrors the spore-quality density for both fast (magenta) and slow (cyan) sporulation. Bottom panel: In poor medium ($q_m = 0$), outgrowth of high-quality spores is strongly favored. These are present for fast sporulation (magenta) but are missing in slow sporulation (cyan). All other parameters as in Fig. S1b.

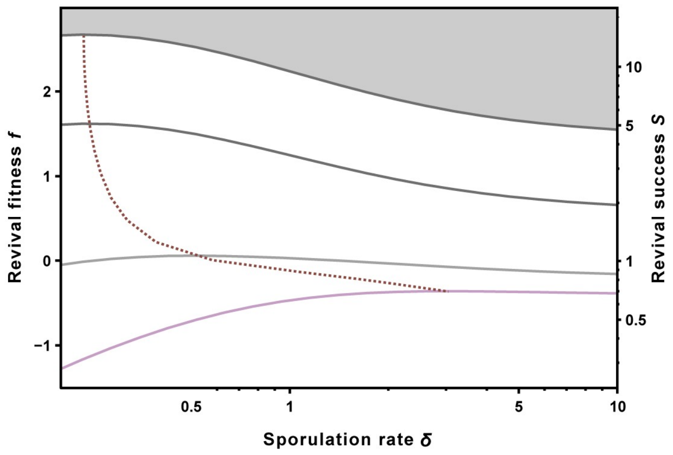

Figure S4. Revival fitness. Revival fitness f *vs.* sporulation rate δ in revival media of increasing richness ($q_m = 0, 0.05, 0.5, 50$, bottom to top, solid lines). The dashed line indicates the optimal sporulation rates over this range. In very rich media, revival success equals yield, $S \rightarrow Y$, so that slow sporulation becomes optimal; the shaded region is inaccessible. In poor media, fast sporulation becomes optimal. At intermediate richness, revival is nearly independent of the sporulation strategy. All other parameters as in Fig. S2b.

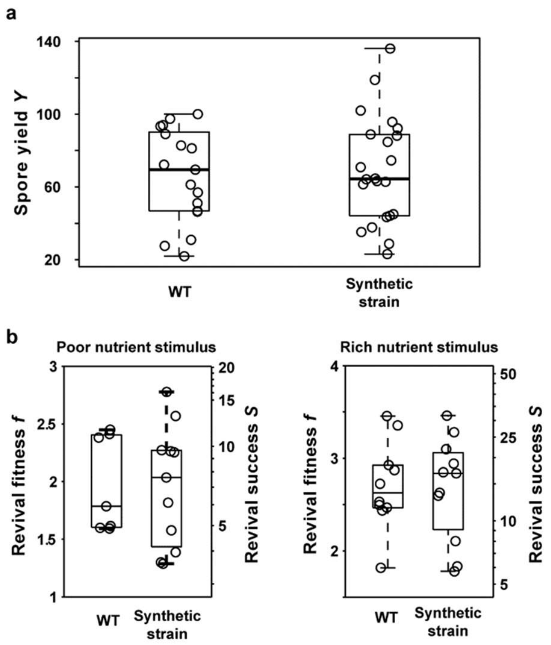

Figure S5. Without IPTG induction the synthetic strain (BIB1332, P_{spank}-*kinA*) and the *wt* (BIB1092) have a comparable spore yield and spore revival fitness. **(a)** Box-plot of the spore yield *Y*. $N \geq 17$ colonies per condition from $n \geq 6$ technical replicates. The differences in spore yield are not significant based on a two-tailed Mann-Whitney U test ($P = 0.05$). $U_Y = 28$, $U_{P=0.05} = 16$. **(b)** Box-plot of the spore revival fitness *f* for a poor nutrient stimulus (left) and a rich nutrient stimulus (right). The differences are not significant based on a two-tailed Mann-Whitney U test ($P = 0.05$). Poor media: $N \geq 7$ colonies per condition from $n \geq 3$ technical replicates, U = 34 ($U_{P=0.05} = 16$). Rich media: $N \geq 10$, $n \geq 3$ U = 52 ($U_{P=00.05} = 26$).

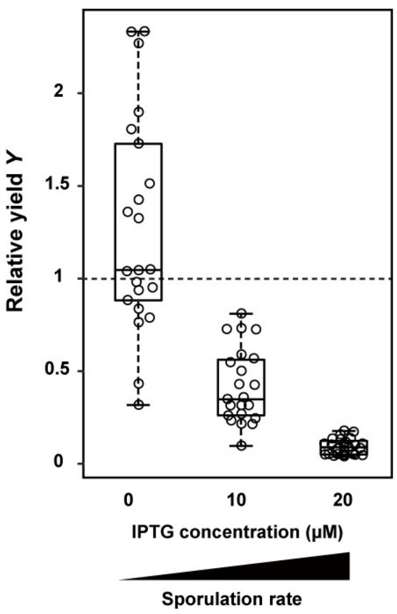

Figure S6. Relative spore yield decreases with increasing IPTG-levels. Boxplot of the relative spore yield $Y = Y_{kinA}/Y_{\mathrm{wt}}$ as a function of IPTG-levels. $N \geq 22$ colonies per condition from $n \geq 5$ technical replicates.

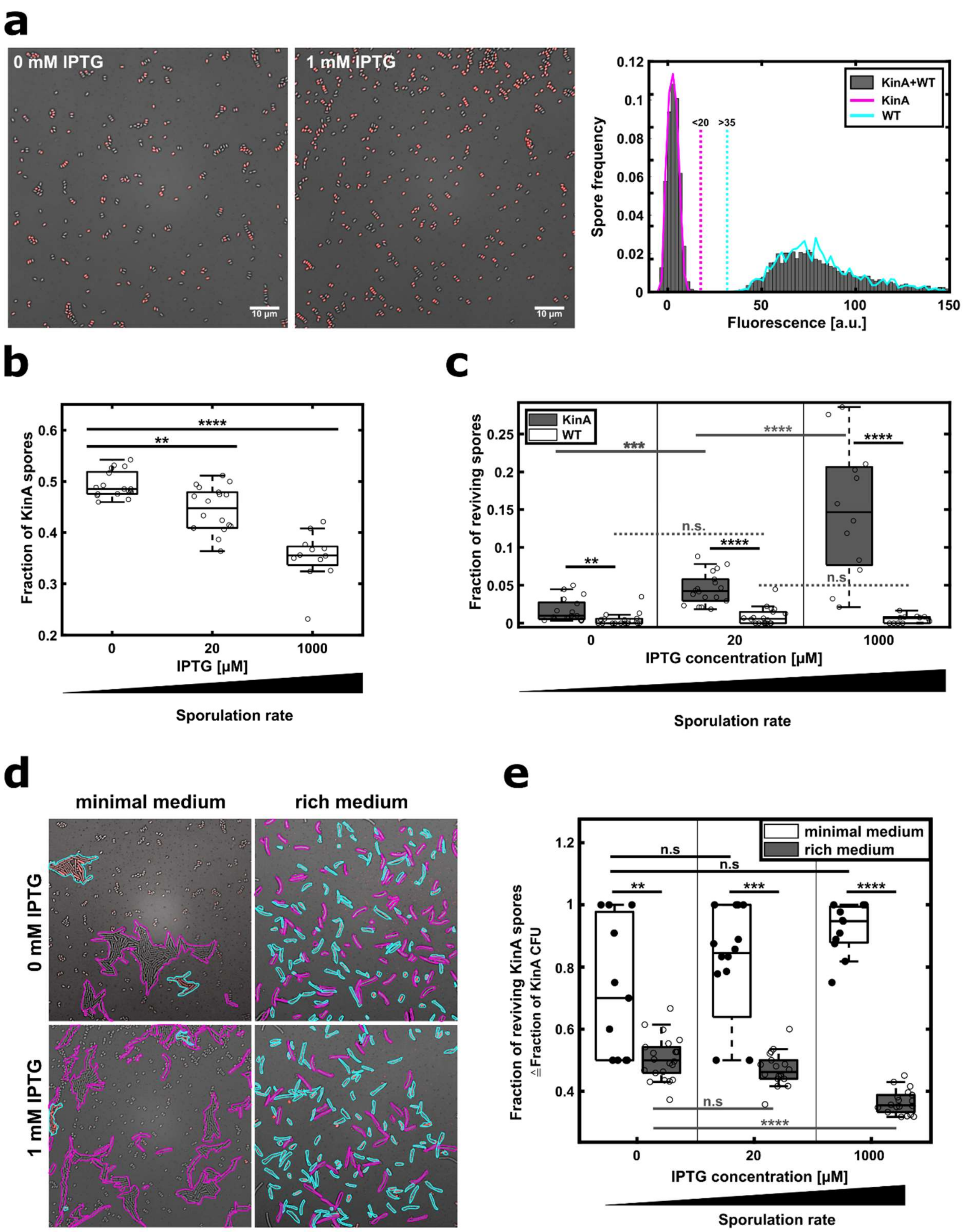

Figure S7. A quality and a yield strategist mixed in liquid sporulation culture exhibit differential revival fitnesses in a nutrient-rich (poor) environment. (a) A synthetic inducible quality strategist (P_{spank}-*kinA*) was mixed with a labeled wt (P_{trpE}-*mCherry*) at a 1:1 ratio in liquid sporulation media. The resulting spores can be distinguished based on their fluorescence. Left: Micrographs of spores derived from a co-culture with and without IPTG induction of *kinA*. The brightfield image was overlaid with the fluorescent image. Right: Histograms of the spore fluorescence distributions of *kinA* (magenta) and wt

(cyan) spores grown in mono-culture are clearly separated and are shown in an overlay with the histogram from a co-culture of both strains (grey). Spores with a fluorescence level lower than 20 counts (higher than 35 counts) were assigned to the *kinA* (wt) strain, respectively. Spores that could not be unambiguously assigned (20 < fluorescence < 35) were excluded from the subsequent analysis. **(b)** The induced quality strategist (*kinA*) produces fewer spores than the yield strategist (wt). Box-plot of the fraction of *kinA* spores [measured by microscopy using spore samples as described in (**a**)] as a function of IPTG. Without induction, the frequencies of *kinA* and wt spores are equal. With increasing IPTG the frequency of *kinA* spores decreases. **(c)** Spores of the quality strategist (*kinA*) have a higher capacity for revival when induced with L-alanine. Spores were dispersed on a SM-pad and stimulated with L-alanine, and spore outgrowth was followed by time-lapse microscopy. Box-plot of revival frequencies of wt and *kinA* spores. Higher IPTG concentrations during sporulation increase the revival frequency of the quality strategist (*kinA*) but have no effect on the yield strategist (wt). **(d, e)** The quality strategist outcompetes the yield strategist in a nutrient-poor environment, while the reverse is true in a nutrient-rich environment. To simulate a nutrient-poor (rich) environment, the formation of microcolony-forming units (mCFUs) from individual spores was monitored on SM pads following the addition of 100 mM L-alanine (a LB-germinant-mix: 33% LB, 33% AGFK, 33 mM L-alanine). The spore mixtures described in (**a**) were used. **(d)** Micrographs of microcolonies in a nutrient-rich (poor) environment after 3 h (16 h), respectively. mCFUs are highlighted in magenta (*kinA*) and blue (wt). Results are shown for spore mixtures produced without IPTG (top) and upon induction of the synthetic quality strategist (*kinA*) with 1 mM IPTG (bottom). **(e)** Box-plot of the fraction of *kinA* mCFUs in a nutrient-rich (full circles) and poor (empty circles) environment, as a function of IPTG levels. mCFUs were approximated by counting the number of outgrowing spores. Statistical information: Each data-point in the box-plots corresponds to data from a single image or movie with > 60 spores in total. Statistical test: Two-sided Mann-Whitney U test, * $p < 0.05$, ** $p < 0.01$, *** $p < 0.001$, **** $p < 0.0001$.

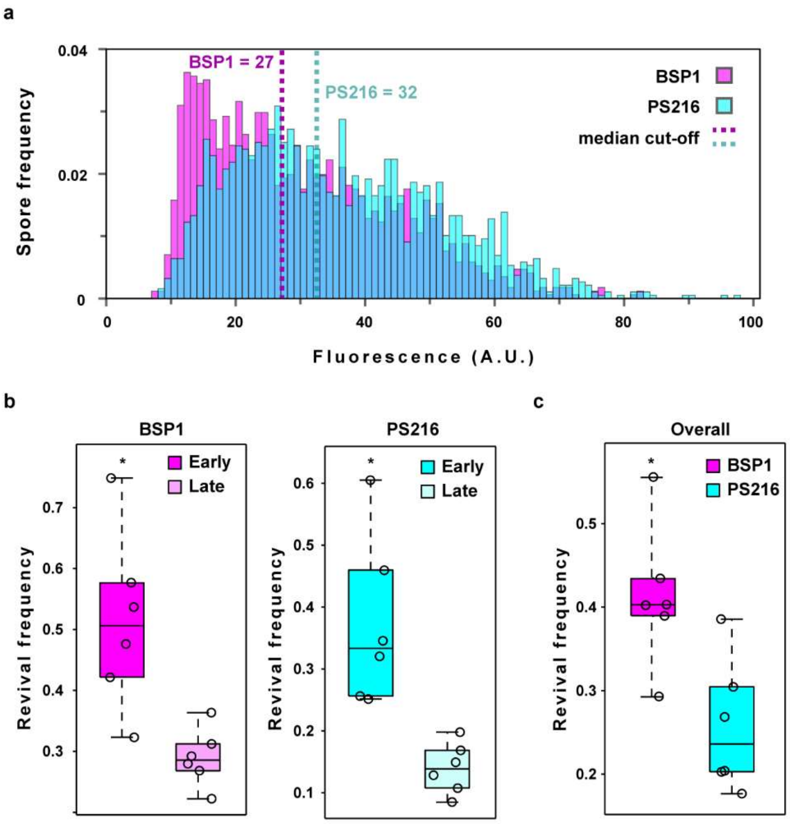

Figure S8. Evidence for a quality-quantity tradeoff in natural isolates. (a) PS216 and BSP1 sporulate heterochronously. Frequency distribution of spore fluorescence intensities. BSP1 (magenta) and PS216 (cyan) were transformed with the spore history reporter (P_{rapA}-*mCherry*). The *rapA* promoter is activated in cells that delay sporulation, therefore late-generated spores have a higher fluorescence. Spores were generated in a shaken culture with SM media. For each strain, the median in spore fluorescence was used to group spores into early-generated (low fluorescent) spores and late-generated (high fluorescent) spores, respectively, in order to test whether sporulation timing affects their revival capacity. **(b)** Box-plot of spore revival frequency for early and late spores as defined in a. Spores were dispersed on a SM-pad and stimulated with L-alanine and the revival frequency was measured by timelapse microscopy. Each data-point corresponds to data from a single movie with on average more than 200 spores in total. For both strains, early spores have a significantly higher revival frequency than late spores ($P = 0.05$, two-tailed Mann-Whitney U test), suggesting that sporulation timing controls the quality of the resulting spores. Statistics: $N = 6$ movies, $U_{P=0.05} = 5$, BSP1: U=1, PS216: U = 0. **(c)** Revival frequency of BSP1 and PS216 spores. Consistent with a higher frequency of very early-forming, high quality spores (see a), a population of spores of BSP1 have a significantly higher overall revival frequency than spores of PS216. Statistical test: two-tailed Mann-Whitney U test ,$P=0.05$, $N = 6$ movies, $U_{P=0.05} = 5$, U=2).

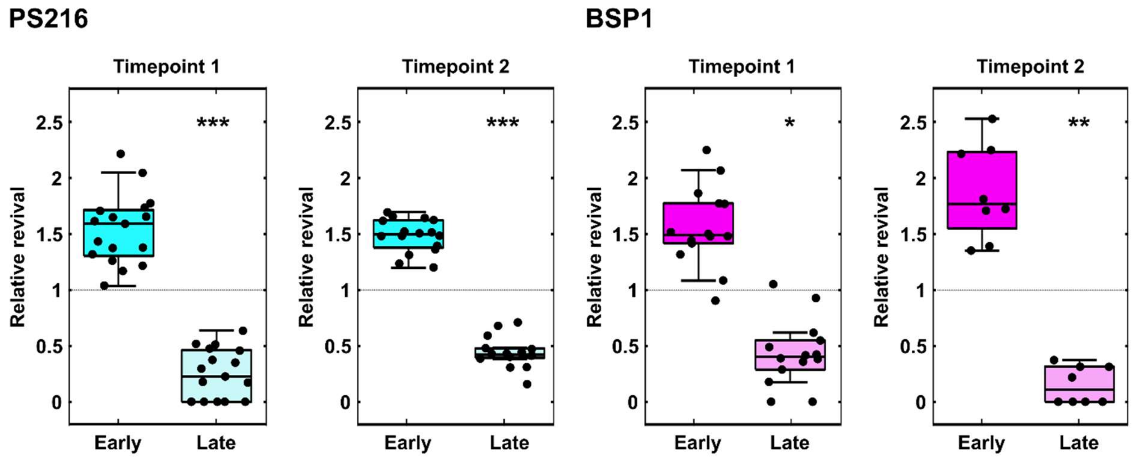

Figure S9. Differences in revival capacity between early and late spores persist when spore age is increased. Box-plot of relative spore revival frequency of fresh (Timepoint 1) and aged (Timepoint 2) spores of the natural isolates PS216 and BSP1. Strains were sporulated in well-mixed liquid culture in microplates and harvested after 2 and 4 days (PS216) or 4 and 10 days (BSP1) of incubation in the sporulation medium. Both strains carry the spore history reporter (P_{rapA}-*mCherry*) that allows one to distinguish early from late spores (time of sporulation) independently of spore age (time that spores have spent in the sporulation medium). The median spore fluorescence was used to classify spores into early and late spores. Spore samples were heat-treated and dispersed on SM pads and stimulated with L-alanine. The revival frequencies for both groups (early and late) were measured by time-lapse microscopy. Movies in which less than 2% of the total spore population grew out were discarded from the analysis. Each data-point corresponds to data from a single movie with > 60 spores in total. To account for technical variations in measurements of the overall revival frequency, the revival frequencies of *early* and *late* spores are reported relative to the overall population mean in each movie. Statistics: $N > 8$ movies. For both strains, early spores exhibited a significantly higher relative revival frequency than late spores, independently of spore age. Statistical test: Two-sided Wilcoxon signed rank test. * $p < 0.05$, ** $p < 0.01$, *** $p < 0.001$.

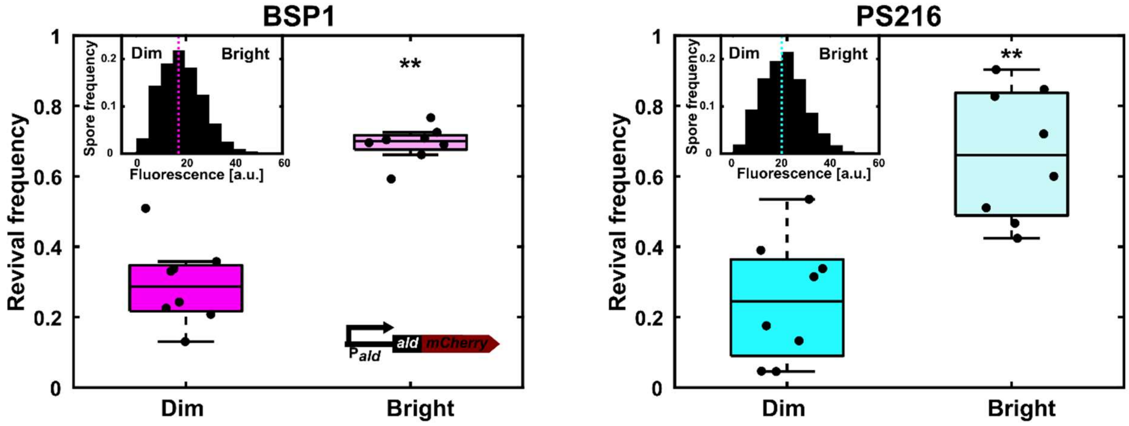

Figure S10. Ald may act as a spore quality determinant in natural isolates. Results are shown for natural isolates BSP1 (left) and PS216 (right) carrying an ectopically integrated fluorescence reporter of Ald protein levels (P_{ald}-*ald-mCherry*). Spores were generated in a well-mixed liquid culture. The insets show the corresponding histograms of spore fluorescence for each strain, which indicate that spores from both strains contain Ald. The median spore fluorescence was used to group spores into "dim" and "bright" classes, which correspond to spores with low and high Ald levels, respectively. Box-plots of the revival frequencies of dim and bright spores are depicted in the main panels. Spores were dispersed on a SM pad and stimulated with L-alanine, and the revival frequency was measured by time-lapse microscopy. Each data-point corresponds to a single movie with > 70 spores in total. Statistics: $N > 7$ movies. For both strains, bright spores had a significantly higher relative revival frequency than dim spores. Statistical test: Two-sided Wilcoxon signed rank test. **$p < 0.01$.

SUPPLEMENTARY MOVIES

Movie S1 (http://doi.org/10.5446/42828**).** Life cycle assay of a yield strategist (BIB 1092: B168 expressing mCherry) and a synthetic quality strategist (BIB1332, *amyE::Pspank_kinA*). Sporulation was induced on SM* pads. Sporulation in the synthetic strain was accelerated by induction of *kinA* with 20 µM IPTG. Spore revival was induced by supplying rich nutrients (LB). For better visibility, the revival response is played in slow-motion.

Movie S2 (http://doi.org/10.5446/42829**).** Life cycle assay of a yield strategist (BIB 1092: B168 expressing mCherry) and a synthetic quality strategist (BIB1332; *amyE::Pspank_kinA*). Sporulation was induced on SM* pads. Sporulation in the synthetic strain was accelerated by induction of *kinA* with 20 µM IPTG. Spore revival was induced by supplying poor nutrients (L-alanine). For better visibility, the revival response is played in slow-motion.

Movie S3 (http://doi.org/10.5446/46818**).** Revival assay of a yield strategist (BIB 444; wt B168 *ppsB::PtrpE-mcherry*) and a synthetic quality strategist (BIB1332; *amyE::Pspank_kinA*) in a nutrient-poor environment. Strains were mixed in a 1:1 ratio in a well-mixed liquid sporulation culture containing 1 mM IPTG to induce *kinA* expression (not shown). Spore revival of the resulting spore mixture was induced on SM pads with 100 mM L-alanine. This nutrient-poor environment selects for spore revival of high-quality spores. Thus, although the quality strategist (*kinA*) produces fewer spores in liquid culture, it generates a larger number of microcolony-forming units (mCFUs) than the yield strategist (wt). mCFUs of the quality (yield) strategist are labeled magenta (cyan). Hence, a nutrient-poor revival environment selects for a spore quality strategy (*kinA*).

Movie S4 (http://doi.org/10.5446/46819 **).** Revival assay of a yield strategist (BIB 444; wt B168 *ppsB::PtrpE-mcherry*) competing against a synthetic quality strategist (BIB1332; *amyE::Pspank_kinA*) in a nutrient-rich environment. Strains were mixed in a 1:1 ratio in a well-mixed liquid sporulation culture containing 1 mM IPTG for induction of *kinA* (not shown). Spore revival of the resulting spore mixture was induced on SM pads with an LB-germinant mix (33% LB, 33% AGFK, 33 mM L-alanine). In this nutrient-rich environment almost all spores revive, regardless of their quality. Since the spore yield strategist produces more spores than the spore quality strategist, it generates more microcolony-forming units (mCFUs). mCFUs of the quality (yield) strategist are labeled magenta (cyan). Hence, a nutrient-rich revival environment selects for a spore quantity strategy (wt).

Movie S5 (http://doi.org/10.5446/42830). Life cycle assay of a *B. subtilis* gut isolate (BSP1, BIB1046) and a soil isolate (PS216, BIB1249). PS216 carries a P_{rapA}-*mCherry* fusion to distinguish it from BSP1. Sporulation was induced on SM* pads. Spore revival was induced by supplying rich nutrients (LB plus AGFK). For better visibility, the revival response is played in slow-motion.

Movie S6 (http://doi.org/10.5446/42831). Life cycle assay of a gut isolate (BSP1, BIB1046) and a soil isolate (PS216, BIB1249). Sporulation was induced on SM* pads. Spore revival was induced by supplying poor nutrients (L-alanine). For better visibility, the revival response is played in slow-motion. Note that reviving spores are more likely to be formed earlier during the starvation period. For BSP1: Out of eleven reviving spores, eight were generated before the median sporulation time (10 h). For PS216: All five reviving spores were formed before the median sporulation time (19 h).

Movie S7 (http://doi.org/10.5446/42832). Life cycle assay of a BSP1 (BIB1048) and a *rapEK*+ (BIB1050, i.e. BPS1 with ectopically integrated *amyE*::*rapE-phrE* and *thrC*::*rapK-phrK* signaling operons) strain. BIB1048 expresses CFP to distinguish it from the mutant strain. Sporulation was induced on SM* pads. Spore revival was induced by supplying rich nutrients (LB plus AGFK). For better visibility, the revival response is played in slow-motion.

Movie S8 (http://doi.org/10.5446/42833). Life cycle assay of a BSP1 (BIB1048) and a *rapEK*+ (BIB1050, i.e. BPS1 with ectopically integrated *amyE*::*rapE-phrE* and *thrC*::*rapK-phrK* signaling operons) strain. BIB1048 expresses CFP to distinguish it from the mutant strain. Sporulation was induced on SM* pads. Spore revival was induced by supplying poor nutrients (L-alanine). For better visibility, the revival response is played in slow-motion.

SUPPLEMENTARY TABLES

Supplementary Table S1. Strains used in this work

Strains	Name	Genotype	Source
E. coli			
	DH5α	*F$^-$ φ80lacZΔM15 Δ(lacZYA-argF)U169 recA1 endA1 hsdR17(r_k^-, m_k^+) phoA supE44 thi-1 gyrA96 relA1 λ$^-$*	Invitrogen
B. subtilis			
**BIB224**	168 1A700	*trpC2*	Laboratory stock (originally obtained from Oscar Kuipers)
**BIB1046**	BSP1		[8], DSM Nutritional Products
**BIB1044**	PS216 3A36		[9, 10] Ines Mandic-Mulec
**BIB182**	168 P*trpE*-mcherry* P*spo0F-yfp* / JL028	*amyE*::[P*spo0F*^{B168}*-yfp cat*^{R}] *ppsB*::[P*trpE*^{*B168}*-mCherry ery*^{R}]	[11]
**BIB1332**	168 P*spank-kinA*	*trpC2 amyE*::[P*spank-kinA*^{B168} *lacI spec*^{R}]	BIB224 transformed with EIB419
**BIB1092**	168 P*hyperspank-mCherry*	*trpC2 amyE*::[P*hyperspank-mCherry lacI spec*^{R}]	BIB224 transformed with EIB424
**BIB1048**	BSP1 P*spoIIG-cfp*	*amyE*::[P*spoIIG*^{BSP1}*-cfp nm*^{R}]	[8]
**BIB1050**	BSP1 *rapEK+*	*amyE*::[*rapE-phrE*^{B168} *cat*] *thrC*::[*rapK-phrK*^{B168} *ery*^{R}]	[8]
**BIB1249**	PS216 P*rapA*II-*mCherry*	*amyE*::[T*gyrA* P*rapA*II^{B168}-*mCherry cat*]	BIB1044 transformed with EIB432
**BIB1282**	BSP1 P*rapA*II-*mCherry*	*amyE*::[T*gyrA* P*rapA*II^{B168}-*mCherry cat*]	BIB1046 transformed with EIB432
**BIB2115**	PS216 P*ald-ald-mcherry* P*ald-gfp*	*amyE::*[P*ald-ald-mcherry cat*^{R} T*gyrA* P*ald-gfpmut3*]	BIB1044 transformed with EIB499
**BIB2117**	BSP1 P*ald-ald-mcherry* P*ald-gfp*	*amyE::*[P*ald-ald-mcherry cat*^{R} T*gyrA* P*ald-gfpmut3*]	BIB1046 transformed with EIB499

**BIB444**	168 P*trpE*-mcherry*	*trpC2 ppsB::*[P*trpE*-mcherry* *ery*^{R}]	[1]
**BIB1440**	168 P*spank-kinA* P*ald-ald-mcherry*	*trpC2 amyE::*[P*spank-kinA lacI spec*^{R}] *sacA::*[P*ald-ald-mcherry lacI kan*^{R}]	[1]
**BIB1392**	168 P*rapA*II-*mCherry*	*trpC2 sacA::*[T*gyrA* P*rapA*II-*mcherry kan*^{R}]	BIB224 transformed with EIB492
**BIB1404**	168 P*rapA*II-*mCherry*	*trpC2 sacA::*[T*gyrA* P*rapA*II-*mcherry kan*^{R}]	BIB224 transformed with EIB492
**BIB1396**	168 P*rapA*II-*mCherry* Δald	*trpC2 ald::mls sacA::*[T*gyrA* P*rapA*II-*mcherry kan*^{R}]	BIB1392 transformed with ald KO cassette
**BIB1397**	168 P*rapA*II-*mCherry* Δald	*trpC2 ald::mls sacA::*[T*gyrA* P*rapA*II-*mcherry kan*^{R}]	BIB1392 transformed with ald KO cassette

Supplementary Table S2. Plasmids used in this work

**Accession Number**	**Plasmid**	**Genotype**	**Source**
**EIB422**	pRFP_Star	'*amyE cat* T*gyrA* LICS promoterless *mcherry amyE*' *bla* ColE1 origin	[1]
**EIB303**	pDR111	'*amyE spec*^{R} T*rrnB* $T_0\lambda$ *Phyperspank-MCS lacI amyE' bla* ColE1 origin	David Rudner, Harvard Medical School
	pDR110	'*amyE spec*^{R} T*rrnB* $T_0\lambda$ *Pspank-MCS lacI amyE' bla* ColE1 origin	David Rudner, Harvard Medical School
**EIB432**	pRFP_Star_P*rapAII*	'*amyE cat*^{R} T*gyrA* P*rapAII-mcherry amyE*' *bla* ColE1 origin	[1]
**EIB424**	pDR111_*mCherry*	'*amyE spec*^{R} P*hyperspank-mCherry lacI amyE*' *bla* ColE1 origin	This work
**EIB419**	pDR110_*kinA* / 04F14	'*amyE spec*^{R} P*spank-kinA lacI amyE*' *bla*ColE1 origin	[12]
**EIB492**	pSac-Kan_P*rapA*II-mCherry	'*sacA kan*^{R} T*gyrA PrapA*II-*mCherry sacA*' *bla* ColE1 origin	This work
**EIB7**	pSac-Kan	'*sacA kan*^{R} *sacA*' *bla* ColE1 origin	[6]
**EIB21**	pDG647	pSB119, mls, source of resistance cassette	[7]

Supplementary Table S3. Oligonucleotides used in this work

Primer	Sequence 5' – 3' (Restriction cut-site in bold)	Purpose
**MA24**	**ATAGTCGAC**GAAGGAGGAAATCACATTATG GTTTCC	Amplification of *mCherry* from BIB182 genome
**MA25**	**ATAGCATGC**TTATTTGTACAGCTCATCCATGC CAC	Amplification of *mCherry* from BIB182 genome
**SONSEQ18**	ATGGCAAGAACGTTGCTCGA	Seq pDR111
**SONSEQ19**	TACGTACGATCTTTCAGCCG	Seq pDR111
**ST39**	CTGGTCGGAGATTGGGATGATAG	Check for chromosomal integration into *amyE* locus
**ST40**	AATTTCCATGTTGCGTAAGTCAG	Check for chromosomal integration into *amyE* locus
**ST129**	ATGAGTATTCAACATTTCCGTGTC	Check for absence of single crossover
**ST130**	TTACCAATGCTTAATCAGTGAGG	Check for absence of single crossover
**CK57**	CGTGCAGATGCGAATAC	Check for intact *ldh* locus (fw)
**CK58**	CGCCATGACTTCACTAAC	Check for intact *ldh* locus (rev)
**ST243**	GTCTGGGTGCCTTCATAC	Seq pSac-Kan-P*rapA*II- *mCherry*
**DW119**	CATAATGGATTTCCTTACGCG	Seq pSac-Kan-P*rapA*II- *mCherry*
**ST283**	3 GCTCAATATGTCGTCATTACAGGC	Check for chromosomal integration into sacA locus
**ST284**	GCAGGGCTAATTGCAGATATAGG	Check for chromosomal integration into sacA locus
**MA51**	GATGAAGATCTGCTGACATTG	ald up_f
**MA52**	CGAGCGCCTACGAGGAATTTGTATCGGGAAC CCCTATGATCATATC	ald up_r
**MA53**	CCTATCACCTCAAATGGTTCGCTGGATCTAGG CTATGAGTATGTTCC	ald down_f
**MA54**	CCAAACAGCCTAAAGACTG	ald down_r
**DW34**	CAGCGAACCATTTGAGGTGATAGGGATCCTTT AACTCTGGCAACCCTC	mls fws
**DW35**	CGATACAAATTCCTCGTAGGCGCTCGGGCCG ACTGCGCAAAAGACATAATCG	mls rev

**KN1**	TCATATCAGCGACGTTCTGC	Δald LFH-PCR check
**KN2**	TTATGTCTTTTGCGCAGTCG	LFH-PCR check
**KN3**	AAATGCGGATGGCACATATT	Δald LFH-PCR check
**LA61**	GTTTTGGTCGTAGAGCACACGG	LFH-PCR check

SUPPLEMENTARY REFERENCES

1. Mutlu A, Trauth S, Ziesack M, Nagler K, Bergeest JP, Rohr K, et al. Phenotypic memory in Bacillus subtilis links dormancy entry and exit by a spore quantity-quality tradeoff. *Nat Commun* 2018; **9**: Article number: 69.

2. Setlow B, Shay LK, Vary JC, Setlow P. Production of large amounts of acetate during germination of Bacillus megaterium spores in the absence of exogenous carbon sources. *J Bacteriol* 1977; **132**: 744–746.

3. O'Brien J, Wilson I, Orton T, Pognan F. Investigation of the Alamar Blue (resazurin) fluorescent dye for the assessment of mammalian cell cytotoxicity. *Eur J Biochem* 2000; **267**: 5421–5426.

4. Nagler K, Setlow P, Li Y-Q, Moeller R. High Salinity Alters the Germination Behavior of Bacillus subtilis Spores with Nutrient and Nonnutrient Germinants. *Appl Environ Microbiol* 2014; **80**: 1314.

5. Setlow B, Setlow P. Levels of oxidized and reduced pyridine nucleotides in dormant spores and during growth, sporulation, and spore germination of Bacillus megaterium. *J Bacteriol* 1977; **129**: 857–865.

6. Middleton R, Hofmeister A. New shuttle vectors for ectopic insertion of genes into Bacillus subtilis. *Plasmid* 2004; **51**: 238–245.

7. Guérout-Fleury A-M, Shazand K, Frandsen N, Stragier P. Antibiotic-resistance cassettes for Bacillus subtilis. *Gene* 1995; **167**: 335–336.

8. Serra CR, Earl AM, Barbosa TM, Kolter R, Henriques AO. Sporulation during Growth in a Gut Isolate of Bacillus subtilis. *J Bacteriol* 2014; **196**: 4184–96.

9. Stefanic P, Mandic-Mulec I. Social interactions and distribution of *Bacillus subtilis* pherotypes at microscale. *J Bacteriol* 2009; **191**: 1756–1764.

10. Durrett R, Miras M, Mirouze N, Narechania A, Mandic-Mulec I, Dubnau D. Genome Sequence of the Bacillus subtilis Biofilm-Forming Transformable Strain PS216. *Genome Announc* 2013; **1**: e00288-13.

11. Levine JH, Fontes ME, Dworkin J, Elowitz MB. Pulsed Feedback Defers Cellular Differentiation. *PLoS Biol* 2012; **10**: e1001252.

12. de Jong IG, Veening J-W, Kuipers OP. Heterochronic phosphorelay gene expression as a source of heterogeneity in Bacillus subtilis spore formation. *J Bacteriol* 2010; **192**: 2053–67.
